# Maternal nutritional status and child feeding practices: a retrospective study in Santal communities, Birbhum District, West Bengal, India

**DOI:** 10.1186/s13006-020-00262-3

**Published:** 2020-05-29

**Authors:** Caroline Katharina Stiller, Silvia Konstanze Ellen Golembiewski, Monika Golembiewski, Srikanta Mondal, Hans Konrad Biesalski, Veronika Scherbaum

**Affiliations:** 1 Institute of Nutritional Sciences, University of Hohenheim, Garbenstr. 30, 70599 Stuttgart, Germany; 2 Shining Eyes -medical aid for children and socioeconomic village development in India e.V., Flein, Germany; 3 Institute of Household and Consumer Economics (530A), University of Hohenheim, Stuttgart, Germany; 4 Bolpur Manab Jamin, South Jambuni, Birbhum District, Bolpur, West Bengal India

**Keywords:** Breastfeeding, Complementary foods, Infant/child feeding, Child care, Maternal anemia, Undernutrition, Santals, Adivasi, West Bengal, India

## Abstract

**Background:**

In West Bengal, according to the National Family Health Survey (NFHS-4) 2015-16, undernutrition and anemia are particularly common among scheduled tribe women and children. The purpose of this research is to assess the nutritional status of Adivasi mothers and child feeding patterns, relevant for clinical practice and the design of future preventive actions. These baseline characteristics were obtained in the scope of a feeding trial aimed at improving the hemoglobin concentration of the index child (6–39 months).

**Methods:**

In February 2015, the baseline survey was conducted in 21 tribal villages. In total, 288 mothers and 307 children were recruited for their hemoglobin levels (HemoCue Hb201+), as well as anthropometric indices height/length, weight and MUAC. By questionnaire-based interview aspects on child feeding practices, childcare, family scheduling, and prenatal care were elucidated.

**Results:**

The majority of mothers belong to the Santal tribe (93.8%). Nearly half of mothers suffered from underweight including severe forms (BMI < 18.5: 49.4%), and the majority of mothers were anemic (Hb < 12 g/dl: 86.2%). Similarly, undernutrition was highly prevalent among the index children. Ever breastfeeding was almost universal in the study area (99.6%), with all infants aged < 12 months at the time of the interview still being breastfed. The majority of children were breastfed within the first hour after birth (75.7%), still every third child (32.2%) was deprived of colostrum. Merely 32.9% of infants were exclusively breastfed for 6 months (180 days) according to the recommendations of the WHO/UNICEF. When relating to the proposed complementary feeding (CF) indicator then 89.6% of children have received CF (mainly family foods/biscuits/plain rice) during the first 6 to 8 months, and 46.8% of children aged 6 to 23 months fulfilled the minimum acceptable diet (2 to 3 meals per day and ≥ 4 food groups per day), corresponding to 58.1% among children aged 12 to 23 months versa 25% among infants aged 6 to 11 months.

**Conclusion:**

The maternal nutritional status was poor and showed interrelations with the nutritional status of the index child. Inadequate feeding and caring practices were common. In particular the younger age group (< 12 months) was found at risk of being offered inadequate CF, which needs to be tackled by future programs.

**Trial registration:**

The trial was retrospectively registered at the German Clinical Trials Register on the 1st July 2019 (DRKS00017388).

## Background

Levels of malnutrition and anemia in children under the age of 5 years in India continue to be among the highest in the world [1, 2]. Globally, about half (45%) of all deaths among children under the age of 5 years are directly or indirectly attributable to nutrition-related factors [3]. In India, the rural-poor, among them the scheduled tribes and castes or the illiterate population are mostly affected by undernutrition [4]. Child malnutrition in India finds its origins almost entirely during the first two to 3 years of life, mostly as a result of frequent infections and inappropriate infant feeding and caring practices [5]. Breastfeeding is the ideal food to promote healthy growth and development of infants providing physiological and psychological advantages for both mother and the child [6, 7], and lowering the risk of mortality from infectious disease in the first 2 years of life [8]. Breastfeeding has been reviewed to have a pivotal role in preventing child malnutrition [9]. The Indian government has always been promoting the global recommendations for optimal infant and young child feeding (IYCF) suggesting an early initiation of breastfeeding, exclusive breastfeeding (ExcBF) for the first 6 months of life, and introduction of CF thereafter with continued breastfeeding up to 2 years, which is in line with the Indian tradition of prolonged breastfeeding and introduction of CF from 6 months of child’s age in the scope of a widely celebrated “annaprashan” -first rice-eating ceremony- commonly found in West Bengal [10].

According to the National Family Health Survey (NFHS-4) 2015-16 [11] ever breastfeeding is almost universal in West Bengal, however still only every second child (52%) under 6 months is exclusively breastfed. Similarly, the initiation of breastfeeding within the first day of life is performed in the majority of children (98%), but merely half (48%) of all children are put to the breast within the first hour of life as recommended. 11% of children received pre-lacteal feedings during the first 3 days postpartum. Breastfeeding is continued at 1 year (97%), up to 2 years (92%) by the majority of mothers. Complementary feeding frequently begins too late, with merely half of children (52%) receiving breastmilk and complementary foods (CF) at age 6 to 8 months. Foods fed to the infant are often nutritionally inadequate or unsafe and match the recommended age-related (6–23 months) minimum number of two to three feedings of solid or semi-solid foods per day in merely 38% of cases, and the same small proportion received foods embracing the four recommended food groups.

Literature review shows that there is limited data available on infant feeding practices among the Santal tribe in West Bengal. In order to obtain more detailed data on the child feeding situation and prevalence of undernutrition among the Santals living around Bolpur, Birbhum District, West Bengal the present study was conducted. Thereby enhanced educational and supplementary strategies can be designed.

## Materials and methods

### Village selection

The registered association Shining Eyes e.V. is providing acute medical aid and preventive village programs to the Santal community in particular from 2011 onwards, with charitable actions already having found its origin in 1994. Presented data was obtained at baseline assessment in the scope of a longitudinal feeding trial (lasting for 1.5 years) initiated by Shining Eyes India in the rural area around Bolpur, Birbhum District. Hereby all 21 villages in the sphere of activity of the Indian non-governmental organization (NGO) Bolpur Manab Jamin (implementing partner) were approached and cluster-randomized into three intervention groups and one control group (the detailed study design and effect analysis will be elaborated elsewhere at a later stage). The starting point of medical checkups in February 2015 provided baseline data before enrolment of participants, which constitute the concern of this paper. All people of the included villages are equally beneficiaries of various government schemes (providing supplement food or antenatal care).

### Target group

The caretakers of all study children did give their informed consent. At baseline assessment prior to applying inclusion criteria of the intervention trial, data on a total of 307 children (aged 6–39 months) and their mothers (*n* = 288) were obtained (it was screened for children aged 6 to 36 months). Hereof ten mothers had two children participating in the study (nine mothers had two singleton children, one mother had twins). Anthropometric and hematological data of pregnant women (*n* = 5) were later on excluded from analysis, similarly data of caretakers who were not the biological mother (*n* = 4). Of total *n* = 297 mothers, *n* = 5 mothers were absent during the 1st checkup due to field work (98.3% was the response rate concerning anthropometric/hematological assessment).

Severely malnourished and/or anemic women and children were referred to the St. Mary’s Child and Mother Health Care Centre for further treatment.

### Sample size calculation

The dependent variable of the intervention study is children’s hemoglobin (Hb) concentration. The estimation of the required overall population proportion resulted in a minimum sample size of *N* = 288 [12]. The confidence level was set at 95%, the margin of error at 0.05, and the estimated prevalence of any anemia in the age group 6 to 35 months for scheduled tribes accounted for 75.3%. The basis for this percentage were data obtained from the NFHS-4, West Bengal, 2015–16 [11]. Hereby the prevalence of anemia in children aged 6 to 59 months was reported with 54.2%, for children aged 6 to 35 months with 61.4%, whereas scheduled tribes 6 to 59 months ranked with 68.1% much higher. The hereby obtained age-related difference of 7.2% was equally assumed for the subgroup scheduled tribes, resulting in an overall expected prevalence of any anemia of 75.3% for tribal children aged 6 to 35 months. The sample size calculation defining the required size of the four different study groups is elaborated elsewhere.

### Anthropometric and hemoglobin measurement and devices

Hb concentration was determined by using a portable hemoglobinometer (HemoCue Hb 201+) for all mothers and children donating a finger prick of capillary blood at each checkup point.

Mid-upper arm circumference (MUAC) was measured to the nearest millimeter using a non-stretchable tape fitting comfortably around the relaxed arm. Thereby the left arm was measured at the mid-point between top of shoulder and elbow (acromion and olecranon).

All subjects were weighed to the accuracy of 0.1 kg by a calibrated seca-floor-scale (seca 877) with 2 in 1 (mother-child function), wearing no shoes and minimal clothes (like sari for mothers, and shirt and shorts for children).

Height was measured for mothers and preferably in the case of children ≥2 years by using seca-mobile stadiometer (seca 217) at the accuracy of 0.1 cm with the subject standing at ground level without shoes. Length measurement was attempted for children < 2 years and was performed by using seca-mobile-mat (seca 210) at the accuracy of 0.5 cm.

The child’s date of birth was obtained from the birth certificate.

In this trial the World Health Organization (WHO) Anthro software 2011 [13] served to compute the stunting, underweight, wasting, body mass index (BMI)-for-age and mid-upper arm circumference (HAZ, WAZ, WHZ, BAZ, and MUAC) scores with the 2006 WHO child growth standard as reference [14]. The interpretation of MUAC (cm) or BMI was performed according to cutoffs adopted by the WHO and joint United Nations (UN) statement [15], or in case of adult MUAC interpretation of additional research has been considered [16–19]. For Hb levels cutoffs by WHO were applied [20].

### Household survey on socio-demographic characteristics and child feeding practices

The initial preparation of the semi-structured questionnaire was based on a set of sample questionnaires having been already successfully applied in other field research in developing countries. These basic questions were supplemented and optimized according to the present study purpose. Precedingly obtained socio-cultural field experience (in terms of longstanding development cooperation of coordinative researchers with the target population) contributed to a high quality and in-depth questionnaire. The pre-testing of the questionnaire was performed in ten Santal households, belonging to two Santal villages in the sphere of activity of the NGO Shining Eyes –and was not part of the later study area. The pre-testing resulted in restructuring and shortening of the draft questionnaire.

Later on the face-to-face interviews with the mothers were conducted by 11 social workers through house to house visits by individual scheduling between Dec 2014 to Apr 2015. The workers received a 3 day training by research authorities as well as by the field-coordinator of the NGO Manab Jamin. During training the whole questionnaire was discussed, practiced by role playing, and finally acquired skills (posing questions in the same way to assure consistent reporting, being non-judgmental etc.) were consolidated in the scope of field exercise in selected households. To draw a holistic picture on the maternal living circumstances of tribal people; information was obtained regarding age, sex, marital status, household (HH) cash income, educational level, family scheduling, antenatal care, delivery mode, as well as IYCF and care. Data quality control was performed in the first weeks of the survey, and in case of uncertainities or contradictory statements the interviewers were sent again to the same HH for further clarifications. Data on prelacteal feeding, initiation of breastfeeding or exclusive breastfeeding (ExcBF) were obtained retrospectively. In regard to ExcBF mothers may have been familiar with the topic “exclusive breastfeeding” as being in touch with Anganwadi Centers –responsible for spreading knowlegde about breastfeeding and IYCF. The awareness of the definition or relevance of ExcBF may have increased self-serving bias. However interviewers were trained to create an athmosphere of trust by meeting the respondents on eye-level, moreover to make clear that all answers are treated anonymously and confidentially. To assess ExcBF rates, mothers were queried if they have given any foods or fluids aside breastmilk during the first 6 months of child’s life (regardless of prelacteal or supplementary feeds during the first 7 days postpartum). If yes, it was clarified if there was any period of time when only breastmilk (not even water) was fed to the child. Subsequently, a further question explicitely asked for the performed or planned (if mother was still exclusively breastfeeding) duration of giving only breastmilk to the child. Thus, the definition of ExcBF was not explicitely stated but commented on in an analogous way, and the term “exclusive breastfeeding” was not included in the question itself, however was contained herein in a descriptive manner.

### Definition of adequate child feeding practices

Proper child feeding practices were defined according to guidelines proposed by the WHO [21]. For the assessment of the minimum meal frequency, mothers were queried to briefly describe a typical daily feeding routine currently practiced, by naming types of foods/meals offered to their child. During analysis foods were categorized in dry foods/snacks that do not need any further preparation (biscuits, cake, bread, chapati/roti, puffed rice, fruits etc.) or cooked foods (Anganwadi Centre/ Integrated Child Development Services (AWC/ICDS) meals, family foods, rice, potato, dal, vegetables, animal products etc.). Milk/breastmilk were not considered in this analysis. The count of provided cooked meals was used to calculate the percentage of children receiving the minimum meal frequency. Moreover mothers were asked if their child had received any vegetables, fruits, or animal products during the previous 24 h, and if yes to specify the type. Together with the current feeding routine of starches and legumes these data were used to define the minimum dietary diversity. Recall bias was seeked to be minimized by preceding training of interviewers, by chronological checking for omitted feeding occasions when assessing the meal frequency and reviewing of the recalled food items. The minimum acceptable diet is composed of the concurrent fulfillment of minimum meal frequency as well as minimum dietary diversity according to age-specific recommendations [21].

### Statistics

The data was coded and analyzed using SPSS statistics 22 (IBM, Armonk, NY, USA). The Kolmogorov-Smirnov (K-S) Test was applied to test for normal distribution, only for less than 50 cases response rate the Shapiro-Wilk test was considered. The general level of significance was set at *p* < 0.05. The results are expressed in percentages or in means ± SD (for non-normally distributed data (according to K-S test) the median is additionally presented). Comparison between groups was performed using the students-t-test or in case of non-normally distributed data the Mann-Whitney test. For the inter-group comparison of multiple groups univariate ANOVA was performed or in case of non-normal distributed data the Kruskal-Wallis test. The Pearson correlation or the Spearman correlation was applied for normal or non-normal distributed data, respectively to elucidate associations between two variables. Sets of categorical data were assessed by Pearson’s Chi-Square test.

## Results

### Socio-demographic characteristics of caretakers of study children

The response rate of the household (HH) baseline survey accounted for 98% (of *n* = 297 HH total). Socio-demographic information (Table 1) addressed, included topics like antenatal care, delivery mode, family spacing, infant and young child feeding practices and care. The main caretaker interviewed was in 98.6% of cases the mother, and in 1.4% the aunt or grandmother due to the reasons that the mother has left the family or died.

**Table 1:** Socio-demographic characteristics of mothers

*Socio-demographic characteristics*	n (%) or Mean ± SD/Median, Min/Max
**Tribe**, (*n* = 291)	
*Santal*	273 (93.8)
*Santal, but converted to Christendom*	1 (0.3)
*Konra*	17 (5.8)
**HH cash income group,** (*n* = 272)	
*0–4999 INR/month*	177 (65.1)
*≥ 5000 INR/month*	95 (34.9)
**Maternal age at birth of first child**^NN***^ (in years, mean ± SD/median, Min/Max), (*n* = 286)	19.9 ± 2.5/19.0, 14/29
**Age group** at birth of oldest child	
*14–17*	29 (10.1)
*18–21*	194 (67.8)
*22–26*	57 (19.9)
*27–29*	6 (2.1)
**Times given birth**^NN***^ (mean ± SD/median, Min/Max), (*n* = 285)	1.8 ± 0.8/2, 1/5
**Times given birth**	
*1*	119 (41.8)
*2*	117 (41.1)
*3*	42 (14.7)
*4*	5 (1.8)
*5*	2 (0.7)
**Birth spacing from pregnancy to pregnancy** (in months, mean ± SD/median, Min/Max)	
*between 1st and 2nd (n = 173)*^NN***^	41.8 ± 24.6/36, 10/186
*between 2nd and 3rd (n = 55)*^NN***^	41.8 ± 23.5/36, 12/99
*between 3rd and 4th (n = 12)*^NN*^	55.9 ± 35.5/45, 12/99
*between 4th and 5th (n = 6)*^NN*^	68.5 ± 36.8/82.5, 24/99
**Use of contraceptives at any time** (*n* = 285)	
*Yes, regularly*	38 (13.3)
*Yes, sometimes*	89 (31.2)
*No*	158 (55.4)
**Mode of delivery** (*n* = 280)	
*natural/vaginal delivery*	264 (94.3)
*cesarean section*	16 (5.7)
**Place of delivery** (*n* = 282)	
*home*	82 (29.1)
*health facility*	197 (69.9)
*on the way to health facility*	3 (1.1)
**Marital status** (*n* = 281)	
*married*	274 (97.5)
*widowed (n = 3), divorced (n = 2), deserted by husband (n = 1), single (n = 1)*	7 (2.6)
**Educational level** (*n* = 288)	
*never attended school*	133 (46.2)
*attended (< 3 years, n = 43), Primary (class V, n = 45), Upper primary (class VIII, n = 37)*	125 (43.3)
*Secondary (class X, n = 23), High school (class XII, n = 6), Above high school (n = 1)*	30 (10.4)
**Daily time** (in hours, mean ± SD, Min/Max) **use on**	
*cooking-related activities (n = 289)*^NN*^	7.8 ± 2.5/7.8, 1/14
*works outside the house/income generating activities (n = 69)*^NN***^	3.4 ± 2.6/2.5, 0.5/8
*child caring activities (n = 240)*^NN***^	2.9 ± 2.0/2.5, 0.5/9.5
*cleaning house (n = 289)*^NN***^	1.3 ± 0.8/1.0, 0.2/4
*cleaning cloth/taking bath (n = 242)*^NN***^	1.3 ± 0.6/1.0, 0.3/3
*relaxing/chatting with friends/sleeping during day time (n = 184)*^NN***^	1.5 ± 0.8/1.0, 0.5/5
all indicated activities during the day (*n* = 290) ^NN***^	14.3 ± 2.2/15, 7/19

*NN* not normally distributed tested by Kolmogorov-Smirnov Test, the hypothesis regarding the distributional form is rejected with *p < 0.05; **p < 0.01; ***p < 0.001

The majority (94.2%) of respondents (*n* = 291) belonged to the Santal community (others belonged to Konra tribe), was married (97.5%), and had schooling below secondary level (89.5%). Nearly every second woman never attended school (46.2%). About half (48.2%) of all women (*n* = 276) would have wished to continue schooling longer than they actually did. Essential reasons for not having continued school (*n* = 143 cases) were firstly parents being incapable to pay for dress, books, and school fees (39.9%), secondly young girls getting married (26.6%), thirdly failure of exams (11.2%), and fourthly the need of girls to earn money for the family (8.4%).

### Anthropometric measurements of mothers and study children

Anthropometric data and hemoglobin levels were obtained from 288 mothers during medical checkup. All in all 307 children participated in the study, 143 (46.6%) females and 164 (53.4%) males, resulting in a sex ratio of 0.872.

At baseline checkup children were aged between 6 and 39 months and suffered from chronic (HAZ -2.03 ± 1.11) and acute (WHZ − 1.19 ± 0.93, MUAC z-score − 1.09 ± 0.88) forms of malnutrition, also reflected in the indicator for underweight (WAZ -1.95 ± 0.98) or BMI-for-age z-score (BAZ − 0.96 ± 0.96). The Hb level ranged from 5.0 to 12.7 g/dl, resulting in an average of 9.1 ± 1.3 g/dl.

Average age of the mothers at the time of the interview was 24.5 ± 3.9 years. Anthropometric measurements of mothers revealed that half (49.4%) of all mothers were suffering from underweight, with one third (33.9%) being classified as mildly underweight, every tenth (10.6%) woman being moderately underweight, and 4.9% being severely underweight. Half of mothers (49.8%) were normal weight, and two women (0.7%) were overweight. According to MUAC more than half were identified as moderately to very severely malnourished (< 23 cm: 59.7%). The majority of women (86.2%) was affected by any anemia, and more than half (60.1%) suffered from moderate or severe forms of anemia.

Table 2 presents selected anthropometric and hematological data of children and mothers.

**Table 2:** Selected anthropometric and hematological characteristics of children and mothers

**Children data**	*n* (%) or Mean ± SD/Median, Min/Max
**Sex ratio**, (*n* = 307)	0.872 (872 girls/ 1000 boys)
**Age** at baseline checkup ^NN***^ mean ± SD/median, Min/Max (months), (*n* = 307)	22.5 ± 9.5/23.0, 6/39
**Age group**, n (%)	
*6 - < 12*	54 (17.6)
*≥ 12 - < 24*	109 (35.5)
*≥ 24 - < 36*	124 (40.4)
*≥ 36 - < 40*	20 (6.5)
**WHZ**, mean ± SD, Min/Max (*n* = 306)	-1.19 ± 0.93, −3.84/2.51
**BAZ**, mean ± SD, Min/Max (*n* = 306)	-0.96 ± 0.96, −3.75/2.87
**HAZ**, mean ± SD, Min/Max (*n* = 306)	−2.03 ± 1.11, −5.75/2.00
**WAZ**, mean ± SD, Min/Max (*n* = 307)	−1.95 ± 0.98, −4.36/1.44
**MUAC z-score**^NN*^, mean ± SD/median, Min/Max (*n* = 307)	−1.09 ± 0.88/− 1.02, − 3.52/1.69
**MUAC**^NN**^, mean ± SD/median, Min/Max (cm) (*n* = 307)	13.8 ± 1.0/13.8, 11/16.8
**Hb**, mean ± SD, Min/Max (g/dl), (*n* = 307)	9.1 ± 1.3, 5.0/12.7
**Maternal data**	
**Maternal age at time of interview**^NN***^ mean ± SD/median (years), Min/Max (*n* = 287)	24.5 ± 3.9/24.0, 18/40
**Height (cm)**, mean ± SD, Min/Max (*n* = 288)	149.5 ± 5.5, 136.4/165.6
**Weight (kg)**^NN*^, mean ± SD/median, Min/Max (*n* = 283)	41.5 ± 4.9/41.1, 31.5/60.0
**BMI (m**^**2**^**/kg)**^NN***^, mean ± SD/median, Min/Max (*n* = 283)	18.6 ± 1.8/18.5, 14.8/27.4
**BMI (m**^**2**^**/kg)**, n (%)	
*Overweight (BMI ≥ 25)*	2 (0.7)
*Normal weight (18.5 ≤ BMI < 25)*	141 (49.8)
*Mild underweight (17 ≤ BMI < 18.5)*	96 (33.9)
*Moderate underweight (16 ≤ BMI < 17)*	30 (10.6)
*Severe underweight (BMI < 16)*	14 (4.9)
**MUAC (cm)**, mean ± SD, Min/Max (*n* = 283)	22.5 ± 1.8, 18.0/29.6
**MUAC (cm)**, n (%)	
*Adequate (23 ≤ MUAC < 32)*	114 (40.3)
*Moderately malnourished (21 ≤ MUAC < 23)*	119 (42.0)
*Severely malnourished (18.5 ≤ MUAC < 21)*	49 (17.3)
*Very severely malnourished (MUAC < 18.5)*	1 (0.4)
**Hb (g/dl)**^NN**^, mean ± SD/median, Min/Max (*n* = 283)	10.4 ± 1.4/10.5, 6.5/14.1
**Hb (g/dl)**, n (%)	
*no anemia (Hb ≥ 12.0)*	39 (13.8)
*mild anemia (11.0 ≤ Hb < 12.0)*	74 (26.1)
*moderate anemia (8.0 ≤ Hb < 11.0)*	153 (54.1)
*severe anemia (Hb < 8.0)*	17 (6.0)

*NN* not normally distributed tested by Kolmogorov-Smirnov Test, the hypothesis regarding the distributional form is rejected with *p < 0.05; **p < 0.01; ***p < 0.001

*Note: n* = 5 pregnant (excluded for analysis of BMI, Hb, MUAC), *n* = 10 mothers have two children participating in the study

Table 3 presents significant correlation analyses between selected mother and/or child study characteristics.

**Table 3:** Correlation between selected study variables to investigate quantitative associations of hematological/anthropometric and nutritional indicators

	Spearman’s Linear Correlation (r)	***p***-value
***Correlation between maternal nutrition indicators***		
*BMI*_*mother*_*x MUAC*_*mother*_*(n = 283)*	0.786**	<0.001***
*BMI*_*mother*_*x Hb*_*mother*_*(n = 283)*	0.128*	0.032*
*MUAC*_*mother*_*x Hb*_*mother*_*(n = 283)*	0.242**	<0.001***
***Correlation between maternal nutrition indicators (BMI, MUAC, Hb) and child hematological or anthropometric data***		
*MUAC*_*mother*_*x MUAC*_*child*_*(n = 293)*	0.332**	<0.001***
*BMI*_*mother*_*x HAZ*_*child*_*(n = 292)*	0.125*	0.033*
*BMI*_*mother*_*x WAZ*_*child*_*(n = 293)*	0.249**	<0.001***
*BMI*_*mother*_*x WHZ*_*child*_*(n = 292)*	0.270**	<0.001***
*BMI*_*mother*_*x BAZ*_*child*_*(n = 292)*	0.242**	<0.001***
*Hb*_*mother*_*x Hb*_*child*_*(n = 293)*	0.175**	0.003**
***Correlation between maternal nutrition indicators (BMI/MUAC/Hb) and child feeding practices***		
*(BMI/MUAC/Hb) x Time of initiation of BF after delivery (categorical) (n = 259)*	(0.062/0.012/0.129*)	(0.321/0.852/0.037*)
*(BMI/MUAC/Hb) x Count of FG consumed (n = 254)*	(0.097/0.133*/0.069)	(0.124/0.034*/0.270)
*(BMI/MUAC/Hb) x Count of FG consumed controlled for child’s age*	(0.072/0.092/0.055)	(0.253/0.144/0.387)
***Correlation between maternal educational level or HH cash income and child feeding practices***		
*(maternal educational level/HH cash income group) x Time of initiation of BF after delivery (categorial) (n = 266/250)*	(−0.110/0.196**)	(0.073/0.002**)
*(maternal educational level/HH cash income group) x Duration of performed ExcBF (months) (n = 230/215)*	(−0.061/− 0.216**)	(0.358/0.001**)
*(maternal educational level/HH cash income group) x Timely introduction of* CF *(at 6th to 8th month, “no/yes* “*question) (n = 250/236)*	(0.043/0.181**)	(0.502/0.005**)
*(maternal educational level/HH cash income group) x Caring time (h/day) (n = 237)*	(0.032/−0.142*)	(0.624/0.034*)
***Correlation between child nutrition indicators and child feeding practices***		
*Hb*_*child*_*x Count of FG consumed (n = 264)*	0.167**	0.007**
*Hb*_*child*_*x Counted FG consumed controlled for child’s age*	*0.094*	0.130

Note: child feeding practices investigated, were having squeezed out any breastmilk before breastfeeding (“no/yes” question), feeding of prelacteal foods (“no/yes” question), time of initiation of BF after delivery (categorial), duration of performed ExcBF (months), timely introduction of CF at 6 to 8 months of child’s life, fulfillment of minimum acceptable diet (“no/yes” question), count of food groups (FG) consumed, count of FG adjusted for age. If associations related to these variables are not presented in Table 3 the correlation was not found to be significant

*Definition of food groups (FG):* starchy foods (grains, roots, tubers), legumes and nuts, dairy products, flesh foods (meat, fish, poultry, organ meats), eggs, vitamin A rich fruits and vegetables, other fruits and vegetables

Asterisks indicate in case of p-values the level of significance p<0.05*, p<0.01**, p<0.001***. Similarly in case of the correlation coefficient r the asterisks indicate if the correlation is significant (p<0.05*) or very significant (p<0.01**) according to the output of SPSS. The strength of correlation is interpreted as follows: up to 0.2 (very weak), up to 0.5 (weak), up to 0.7 (moderate), up to 0.9 (high)

Maternal BMI and MUAC were *highly* correlated (*r* = 0.786**), proofing accuracy of measurements performed as MUAC has been found to adequately predict BMI levels [22]. In contrast, there was merely a *very weak* association (*r* = 0.128*) between the BMI and Hb of mothers, and also a *weak* association between maternal MUAC levels and Hb (*r* = 0.242**), still indicating maternal hemoglobin levels being associated with maternal nutritional status.

The higher the maternal Hb level or HH cash income, the later the mother tended to initiate breastfeeding after delivery. The higher maternal MUAC, the more FG she offered to her child over the day, however this quantitative association turned non-significant when controlling for child’s age.

Moreover, the higher the HH cash income, the shorter the duration of performed ExcBF or the caring time for child-related activities, in turn being associated with a timely introduction of CF at 6 to 8 months of child’s life.

### Mother-child dyad analysis in regard to the variables Hb, MUAC, and maternal BMI versa child’s anthropometric indices

**Fig. 1:**
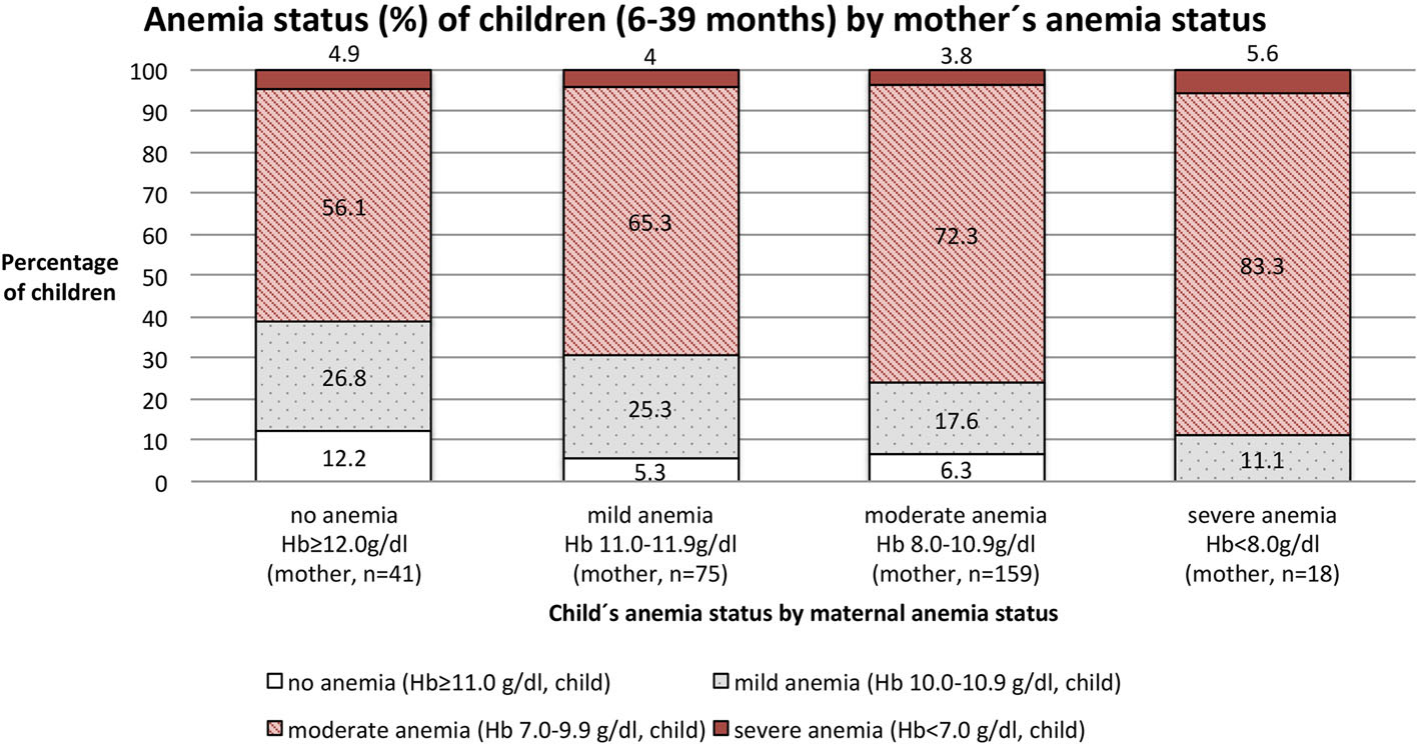
Anemia status of children (*n* = 293) in percent by mother’s anemia status (*n* = 283), with ten mothers having two children participating in the study

The Spearman correlation detected a *very weak* positive association between the hemoglobin of mothers and children (*n* = 293) (*r* = 0.175**). A non-anemic mother (*n* = 41) was more likely to have a non-anemic child and the likelihood of her child suffering from moderate anemia was less as compared to mothers suffering from any form of anemia. The proportion of children suffering from severe anemia seemed to be unaffected by maternal anemia status (Fig. 1). When selecting mothers and children suffering from no anemia/mild anemia versa moderate/severe anemia and testing for independence of the variables, the association proved to be significant (*p* = 0.038).

**Fig. 2:**
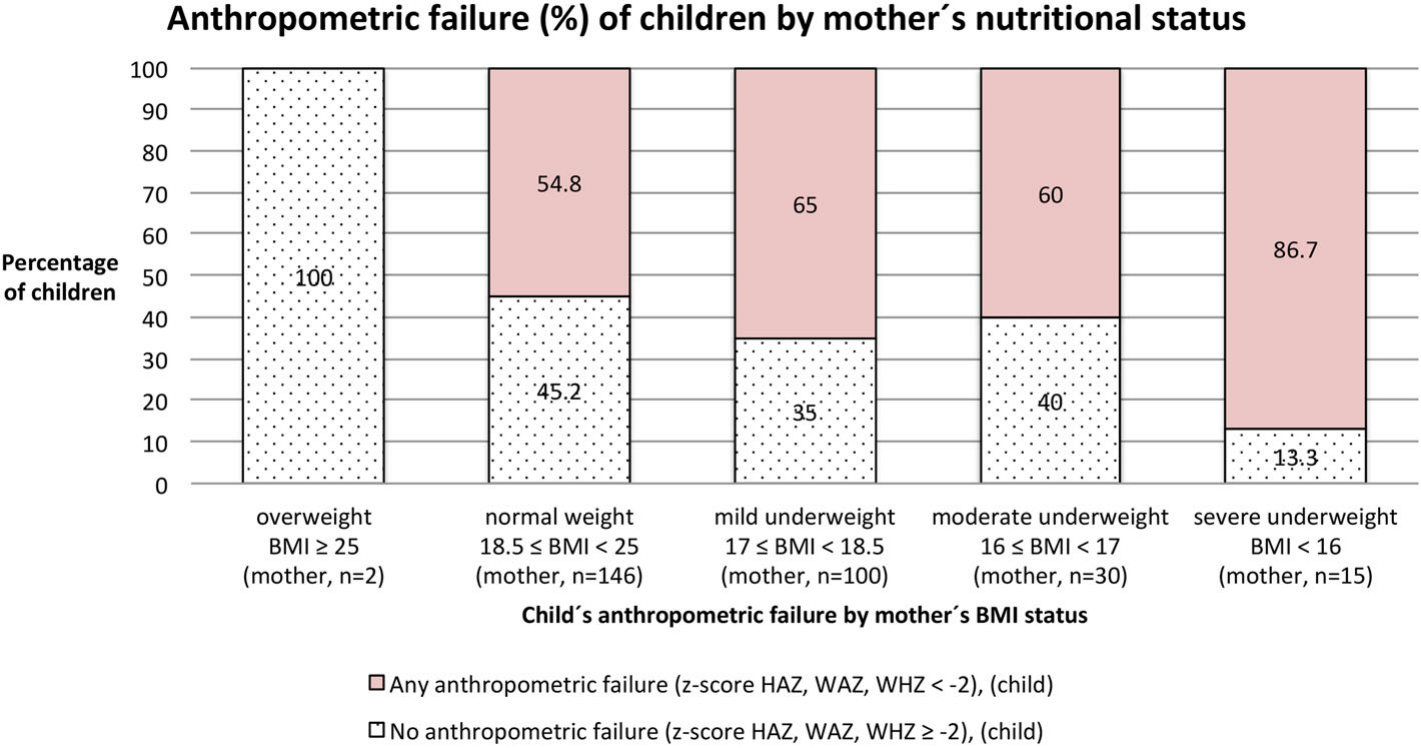
Anthropometric failure of children (*n* = 293) by maternal nutritional status (*n* = 283), with *n* = 10 mothers having two children participating in the study

The nutritional status of mothers is related to the nutritional status of study children (Fig. 2). Children whose mothers are underweight (BMI < 18.5, *n* = 145) were more likely to suffer from single or combined anthropometric failures concerning stunting, underweight, or wasting than children of mothers with an adequate or higher BMI (*n* = 148), *p* = 0.025. Between the MUAC (cm) of children and mothers (*n* = 293) a *weak* positive association (*r* = 0.332**) was detected, similarly the BMI of mothers correlated positively (*r* = 125*, *r* = 0.270**, *r* = 0.242**, *r* = 0.249**) with the HAZ, WHZ, BAZ (*n* = 292) and WAZ (*n* = 293) score of children. Hence, data gathered within this study proves that weak mothers do give birth to weak children, perpetuating the intergenerational cycle of malnutrition.

### Child care

Caretaking of the youngest child (*n* = 281) was in 93.2% of cases the responsibility of the mother herself, 5.0% by the grandparents of the child, 1.8% by mother and father together, 1.1% by older siblings, 0.4% by the aunt of the child. Still, since the birth of the youngest child (*n* = 277), 40.8% of mothers reported to have worked outside the house. When relating to the age of the children at the time of the interview, merely 9.3% of mothers with a child aged 0 to 11 months reported to work outside the house. This percentage was increasing by child’s age: 12 to 23 months (30.6%), 24 to 35 months (64.0%), or ≥ 36 months (53.8%). Thus, it can be concluded that after the first year of the child’s life mothers increasingly worked outside the house, with more than half of mothers pursuing outward works after the child has turned 2 years. Of those working outside the house and specifying child’s stay (*n* = 109 cases) the child was kept at home (81.7%), at the worksite (15.6%), with neighbors or relatives (2.8%), or at the local AWC (0.9%). At the worksite (*n* = 20) the child was left alone in the majority (*n* = 12) of cases, and those children left at home (*n* = 93) were supervised by the grandparents of the child (*n* = 50), elder sibling (*n* = 21), aunt (*n* = 8), husband (*n* = 6), nobody (*n* = 6), mother-in-law (*n* = 2), or neighbors (*n* = 1).

If mother and child are eating together at home (*n* = 279), the mother sits close to the child (88.5%), only sometimes attends the child during eating (8.6%), normally is not sitting with the child (2.2%). Reasons for not sitting close to the child (*n* = 25), were no time/too much work (*n* = 13), mother feels that it is not necessary (*n* = 9), father or grandmother is eating with the child (*n* = 2), child eats independently (*n* = 1). Of all mothers normally sitting next to their child during eating (*n* = 253), the majority of mothers (86.6%) helped their child to eat and every third mother (31.2%) actively encouraged her child to eat by speaking or laughing to the child, less than a tenth (7.5%) of women stated to be eager to feed all food in bowl until finished.

### Time use and activities over the day

For the time when there was no cultivation/harvesting season 268 mothers reported to spend most of their time on cooking (78.7%), looking actively for the child (20.1%), cleaning the house (4.9%), working in the field (3.7%), doing other work outside the house (3.7%), cleaning cloths (2.6%), or fetching water (1.5%) from village pond or tube well. The majority out of 286 mothers (88.8%) felt to have sufficient time to finish all needed works and to look after the child, 5.9% felt that only sometimes sufficient time was left aside work to look after the child, and 5.2% stated that the house and field work don’t leave enough time to look after the child.

When reporting all daily activities, most time is spent on cooking-related activities (*n* = 289, collecting fuel, cow dung cake making/drying as fuel, collecting vegetables, local marketing, hunting, fishing, fetching water, cooking, cleaning utensils, eating together) accounting for 7.8 ± 2.5 h, with a minimum of 1 h up to 14 h dependent on the number of activities routinely pursued per day. On works outside the house (*n* = 69, field work, paid work other than field work, caring for livestock including cleaning the shade, crop drying/storing grains) an average of 3.4 ± 2.6 h was spent daily. Child related activities (*n* = 240, caring for the child, eating with the child, eating at the AWC, helping the children to learn) accounted for an average of 2.9 ± 2.0 h, with a minimum of 0.5 h up to 9.5 h a day. On cleaning cloths or taking bath (*n* = 242), between an average of 1.3 ± 0.6 h was spent, and on cleaning the house (*n* = 289) an average of 1.3 ± 0.8 h. To relax during the day (*n* = 184, sitting together and talking with family, friends, or neighbors, watching TV, sleeping during the day time) a minimum of 0.5 h up to 5 h was spent with an average of 1.5 ± 0.8 h. Indicated daily activities taken together resulted in an average of 14.3 ± 2.2 h, with a minimum of 7 h up to 19 h.

During plantation or harvesting season (*n* = 288) the majority of women indicated to have a maximum of 30 min´ (42.4%), or 1 h to 2 h (35.4%) time to sit, relax and chat together with family members or friends but only 6.3% enjoyed a relaxing time longer than 2 h and 16.0% reported not to have at all time to relax. Outside the plantation or harvesting season (*n* = 289) merely 1.4% indicated to have no time at all left for relaxing, and 12.5% indicated to have a maximum of 30 min time, with the majority reporting to enjoy coming together with family or friends for 1 h to 2 h (55.4%) or longer than 2 h (30.8%).

### Family scheduling, antenatal checkups, delivery mode and use of vitamin or mineral supplements

The age of the mother at the birth of her oldest child (*n* = 286), ranged from 14 up to 29 years, with an average age of 19.9 ± 2.5 years. At the time of the interview the average age of mothers (*n* = 287) was 24.5 ± 3.9 years, and the number of children (*n* = 285) delivered ranged from 1 to 5 with an average of 1.8 ± 0.8 children. 41.8% of mothers had one child, 41.1% two children, 14.7% three children, 1.8% four children, 0.7% five children. Women aged above 30 years (*n* = 19), had on average three children (3.2 ± 1.0, Min/Max: 1/5 children).

Of 282 women, every tenth (11.3%) had lost a child before due to the following reasons (*n* = 31): child death shortly after birth (*n* = 11), stillbirth (*n* = 8), preterm birth (*n* = 7), or illness (*n* = 4), abortion (*n* = 1).

Of 285 women, 13.3% regularly used contraceptives, one third of women (31.2%) sometimes made use of contraceptives, more than half (55.4%) did not use any contraceptive. Of those making use of contraceptives (*n* = 114), the majority (78.9%) took the pill, 16.7% performed tubal ligation, 3.5% of husbands used a condom, and 0.9% just specified that it was a permanent method of family planning. Despite the limited use of contraceptives the time between experienced pregnancies accounted for an average of more than 3 years.

The place of delivery of the youngest child (*n* = 282) was in one third of cases (29.1%) at home, the majority of women (69.9%) delivered at a health facility and 1.1% on the way to a health facility.

The mode of delivery (*n* = 280) was in the majority of cases (94.3%) a natural vaginal delivery, and in 5.7% of cases cesarean section. Complications (*n* = 265) occurred in 5.7% of natural deliveries. The reason for having a cesarean section (*n* = 17), was the doctor decided (*n* = 14), an emergency situation occurred during the process of natural delivery (n = 2), mother wished herself a cesarean section (*n* = 1). The type of delivery assistance specified (*n* = 277) was in the majority of cases a doctor (71.8%), followed by traditional birth attendant (village midwife) (22.0%), a trained birth attendant (trained midwife) (14.4%), and friends or relatives (6.1%).

Of 280 women 1.4% did not go for antenatal care (ANC) in the case of her youngest child, 2.1% had one checkup, 8.2% two checkups, and the majority of cases three (35.0%), four (41.4%), or five to seven checkups (11.8%). Thus, the number of checkups ranged from 0 to 7, with an average of 3.5 ± 1.1. The gestational age when the 1st checkup was performed (*n* = 276), was on average at the 3.4 ± 1.1 month, taking place at a range of the 1st up to the 9th month of gestation.

Almost half (40.1%) of all women (*n* = 289) took supplements or micronutrient powders in the last year. Hereof (*n* = 109), iron (67.9%) and vitamin A (50.5%) were the most common supplements, followed by multivitamin compounds (26.6%), folic acid (20.2%), and zinc (2.8%). About half (55.4%) of all women (*n* = 112) received the supplement from the government health care centre, one third (32.1%) got the supplements from a shop, every tenth woman (9.8%) got it from the pharmacy, followed by AWC (3.6%), registered medical practitioner (2.7%), village quack doctor (2.7%). The reason (*n* = 113) for taking supplements was because of weakness (50.4%), pregnancy (48.7%), sickness (9.7%), breastfeeding (3.5%), clinic told (3.5%), family told (2.7%), doctor told (0.9%), operation-after-care (0.9%).

### Breastfeeding

Ever breastfeeding (as compared to never breastfeeding) was performed for any period of time (*n* = 280) by 99.6%, except one mother who died at birth. Even >though breastfeeding was universally practiced, 14 mothers reported to make use of breastmilk substitutes in addition, due to the perceived feeling of “not having enough breastmilk” (*n* = 13), and one woman being unclear about the reason. Stated substitutes were infant formula (*n* = 8), infant formula & cow milk (*n* = 1), infant formula & cow milk & water (*n* = 1), infant formula & candy water (*n* = 1), cow milk (*n* = 2), cow milk & water (*n* = 1).

At the time of the interview all children aged younger than 12 months (*n* = 53) were still breastfed, and 98.0% of children aged 12–23 months (*n* = 98). Of children aged 24 to 40 months (*n* = 121), 90.1% were still breastfed.

When asked about when to stop breastfeeding their child (*n* = 258), the majority of mothers (81.8%) had “no specific plan”, “when I am pregnant again” (8.1%), “when the child has a specific age” (8.1%), “when I have delivered the next child” (0.8%), “as long my child eagerly continues breastfeeding I will continue” (0.4%), “when child refuses the breast by itself “(0.4%), “mother moved away, thus breastfeeding could not be continued” (0.4%). When asked for the specific age (*n* = 14) at which breastfeeding can be stopped, the average time was indicated at 35.6 ± 7.6 months, ranging from 24 to 48 months.

**Fig. 3:**
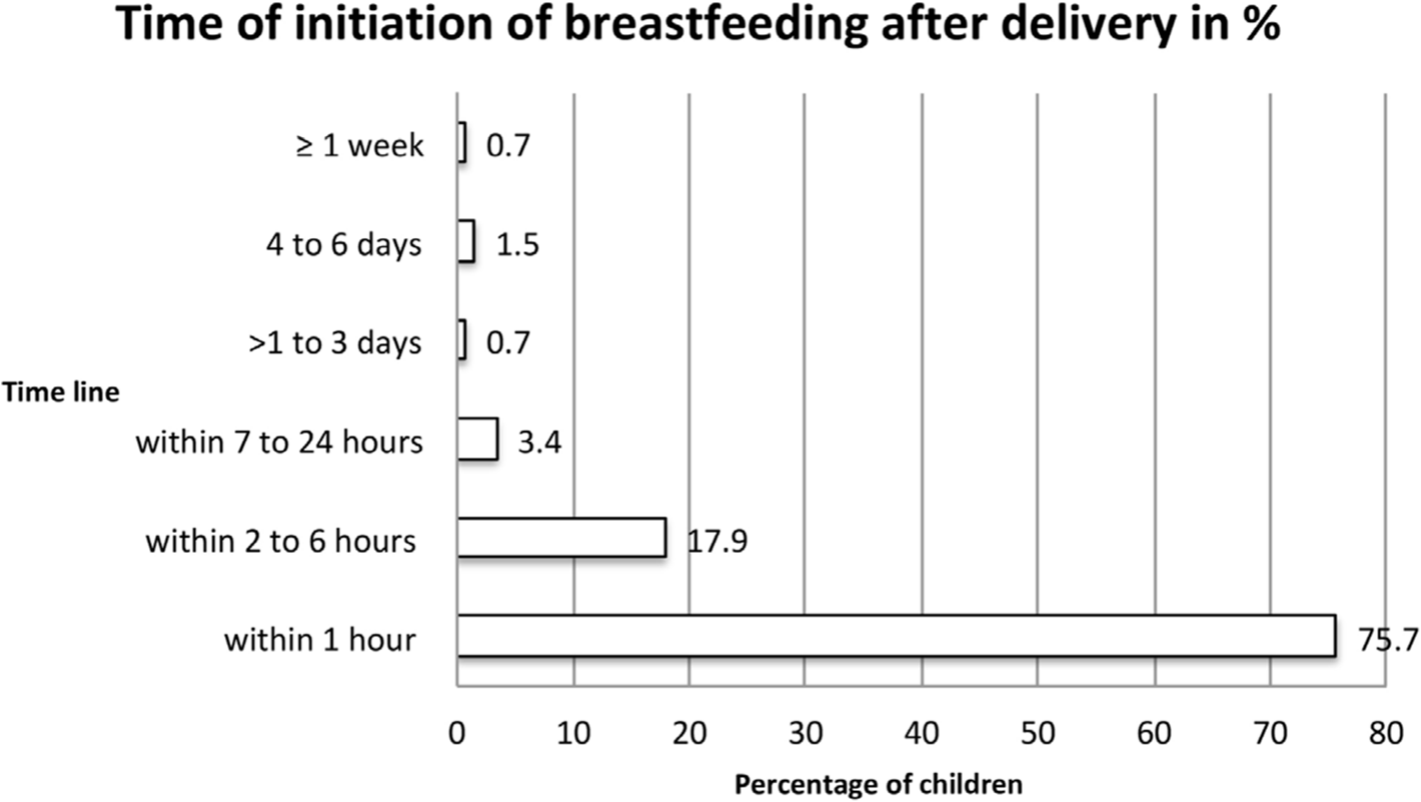
Time of initiation of breastfeeding after delivery (*n* = 268). *Note: n* = 6 women were unclear about that question and their responses are not included in that figure

The retrospectively assessed time point of breastfeeding initiation after birth (*n* = 268) (Fig. 3), was in the majority of cases (75.7%) immediately (within 1 h after birth) as recommended by the WHO/United Nations International Children’s Emergency Fund (UNICEF) [23], followed by 2 to 6 h after birth (17.9%). A smaller proportion of women started breastfeeding within 7 to 24 h after birth (3.4%), > 1 to 3 days after birth (0.7%), 4 to 6 days after birth (1.5%), or ≥ 1 week after birth (0.7%).

The majority (93.2%) of all children (*n* = 278) was not given any liquids or solid foods in the time after delivery before receiving any breastmilk (pre-lacteal feeding*)*. The type of pre-lacteal feedings (*n* = 19), were candy/michri water (*n* = 8), honey (*n* = 6), formula milk (*n* = 3), boiled plain water (*n* = 1), saline due to illness (*n* = 1). The named reasons (*n* = 7), were family tradition (*n* = 4), child was too sick (*n* = 1), mother was sick, thus formula milk was fed (*n* = 1), elders told to give honey (*n* = 1).

Similarly the practice of providing supplementary liquids or solid foods besides breastmilk during the first 7 days postpartum (*n* = 277) was not frequently found in the study area (9.4%). Of those receiving supplementary feeding during the first week after delivery (*n* = 26), the most common supplementary food was formula milk (*n* = 10), honey (*n* = 7), candy/michri water (*n* = 6), boiled plain water (*n* = 3), or cow milk (*n* = 3).

Except the first week after delivery (*n* = 267), the majority of mothers (76.8%) indicated not having provided any foods or fluids to their child besides breastmilk for any period of time during the first 6 months of child’s life (ExcBF). Indicated liquids or foods regularly given to the child aside breastmilk (*n* = 61), were formula milk (*n* = 25), cow milk (*n* = 11), family food (*n* = 9), boiled plain water (*n* = 10), rice (*n* = 8), unboiled plain water (*n* = 4), honey (*n* = 3), biscuits (*n* = 2), candy/michri water (*n* = 2).

**Fig. 4:**
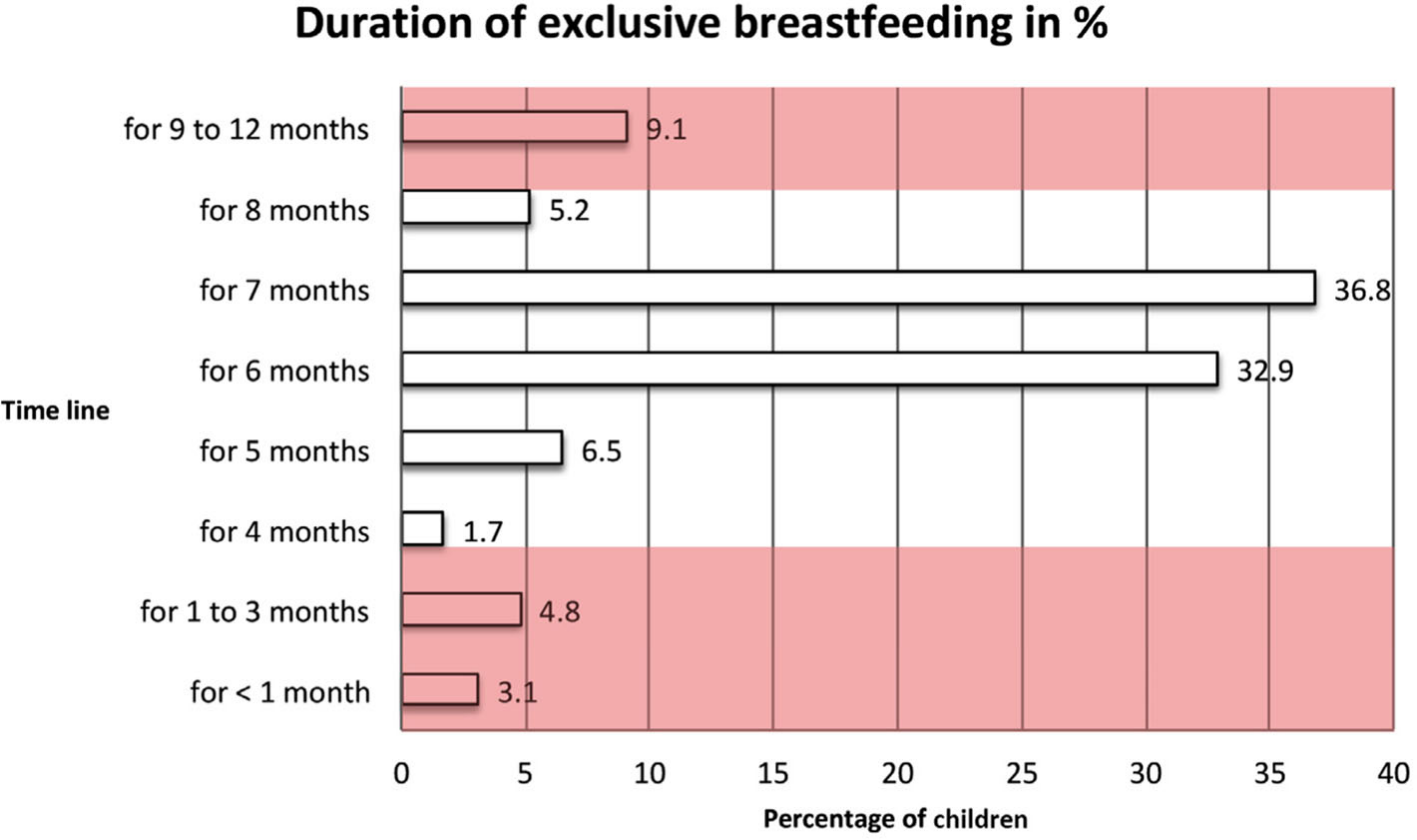
Duration of performed ExcBF (*n* = 231). *Note: n* = 32 women were unclear about that question, *n* = 12 women were still exclusively breastfeeding at the time of the interview, thus their responses are not included in the figure. The planned duration was on average 7.5 ± 3.0 months, ranging from 1 to 14 months. The rationale for the time frame of 4 up to 8 months, indicted as being still acceptable for practicing ExcBF or introduction of complementary foods in the Figs. 4 and 5, is based on the combined interpretation of the proposed feeding indicators by WHO/UNICEF [21], as these two indicators are intertwined for holistic interpretation

The duration of ExcBF^NN***^ (*n* = 231) (Fig. 4), accounted for an average of 6.4 ± 2.0 months (median: 7.0 months), ranging from < 1–12 months.

However, merely one third (32.9%) performed ExcBF for six full months according to the recommendations of WHO/UNICEF [23]. A similar proportion (36.8%) performed ExcBF for seven full months, and 5.2% for 8 months. Still, 16.1% performed ExcBF for less than 6 months, or exclusively breastfed their child too long for a period of 9 up to 12 months (9.1%).

More than half of all children (*n* = 262), received any medicine or micronutrient tablets in the first 6 months of their life (61.1%). The type of medicine (*n* = 104), was medicine against cough (38.5%), vitamin A (34.6%), medicine against fever (30.8%), polio vaccination (16.3%), multivitamin compounds (3.8%), homeopathic medicine (2.9%), saline (hospitalized) (1.0%), medicine for liver problems (1.0%), or enzyme drops (1.0%).

Before breastfeeding the very first time (*n* = 272), one third (32.0%) did squeeze out and discarded their milk (colostrum). Reasons for squeezing out fluid from the breast (*n* = 55), were “elders told” (*n* = 24), “sticky thick breastmilk” (*n* = 6), “breast pain” (*n* = 5), “doctor told to squeeze out yellowish milk” (*n* = 4), “mother told” (*n* = 4), “doctor and nurse told” (*n* = 3), “village midwife told” (*n* = 3), “child was not eating” (*n* = 3), “everyone squeezes out breastmilk as she knows/everyone told” (*n* = 2), “family members told” (*n* = 1), “ICDS worker told” (*n* = 1), “child was very sick” (*n* = 1), “only little” (*n* = 1). When mothers (*n* = 273) were queried if their child has received the yellowish milk the majority (67.8%) of mothers stated that their child had received the colostrum, every fifth mother (22.3%) stated that she had initially expressed some breastmilk but then has fed the remaining yellowish milk to her child, every tenth woman (9.2%) felt not having fed the yellowish milk to her child due to initial expression but was unsure about the extent of expression, 0.7% were sure not having fed the yellowish milk at all. The reasons for especially not having fed the yellowish milk (*n* = 8), were “the subcenter advised to squeeze out first yellow milk”(*n* = 2), “for stickyness” (*n* = 2), “elder advised” (*n* = 2), “child was too sick to be breastfed” (*n* = 1), “child did not eat breastmilk” (*n* = 1).

On demand feeding (*n* = 276), was practiced by the majority of women (84.1%), about one third of all women (*n* = 272) agreed to the statement to offer the breast in regular intervals (32.4%), but more than one third (37.7%) of 273 women reported not to wake up their baby for breastfeeding even if it sleeps several hours. A breastfeeding session is usually stopped (*n* = 272), when the child decides itself to come off the breast (98.2%), in merely 1.8% of cases the mother is deciding when to stop the breastfeeding session. During the night (*n* = 274), the majority of women (98.2%) breastfed their child when aged younger than 6 months, 1.8% reported that the baby sometimes sleeps through the night without feeding. The average number (*n* = 249) of breastfeeding the child during the night accounted for 3.8 ± 1.1 times, ranging from 1 to 7 times. Every tenth woman (12.8%) breastfed 1 to 2 times during the night, the majority (65.8%) had 3 to 4 breastfeeding sessions, and every fifth woman (21.3%) breastfed 5 to 7 times during night’s rest.

### Complementary feeding & young child feeding practices

The majority (93.2%) of all children (*n* = 279) received regular feedings of CF at the time of the interview.

The first regular feedings of soft or solid foods^NN***^ (*n* = 252), were on average at 6.8 ± 1.6 months (median: 7.0 months), ranging from 2 to 12 months.

**Fig. 5:**
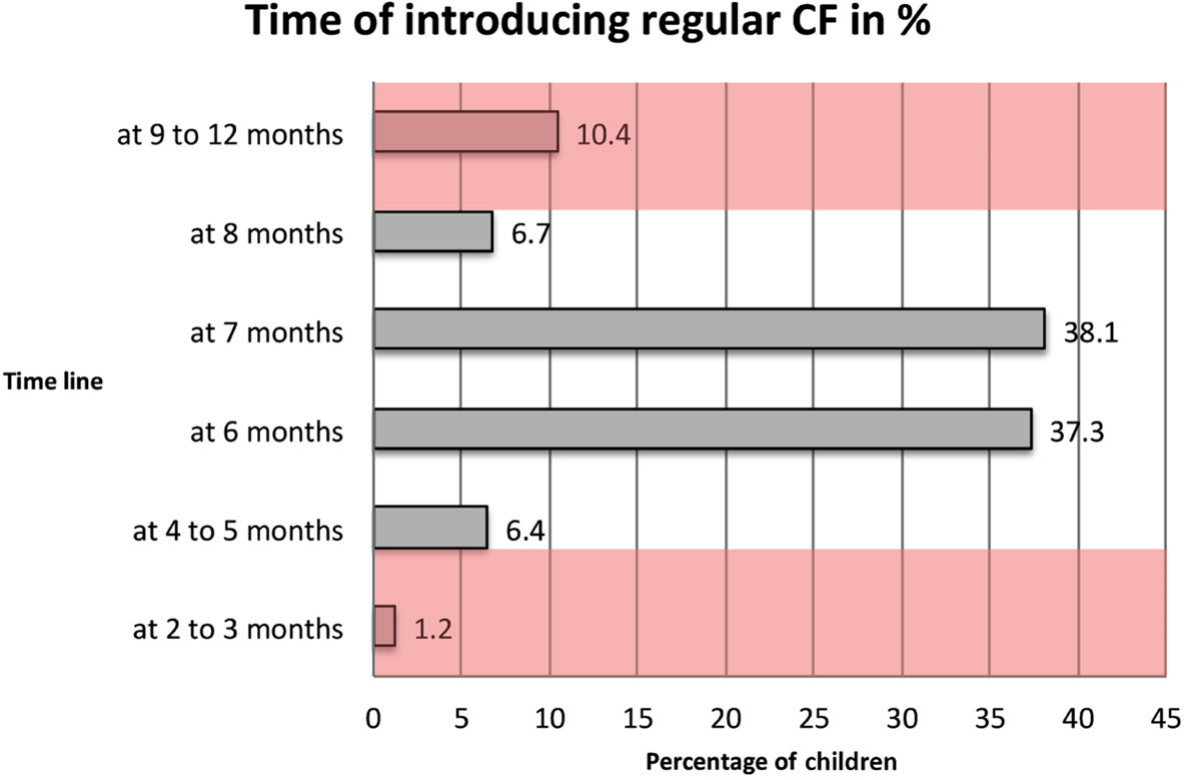
Age of having introduced regular complementary feedings (*n* = 252). *Note: n* = 11 women were unclear about that question, *n* = 17 women had not yet introduced CF, thus their responses are not included in the figure. The planned introduction of CF^NN**^ was on average 7.8 ± 1.9 months (median: 7.0 months), ranging from 6 to 14 months

**Fig. 6:**
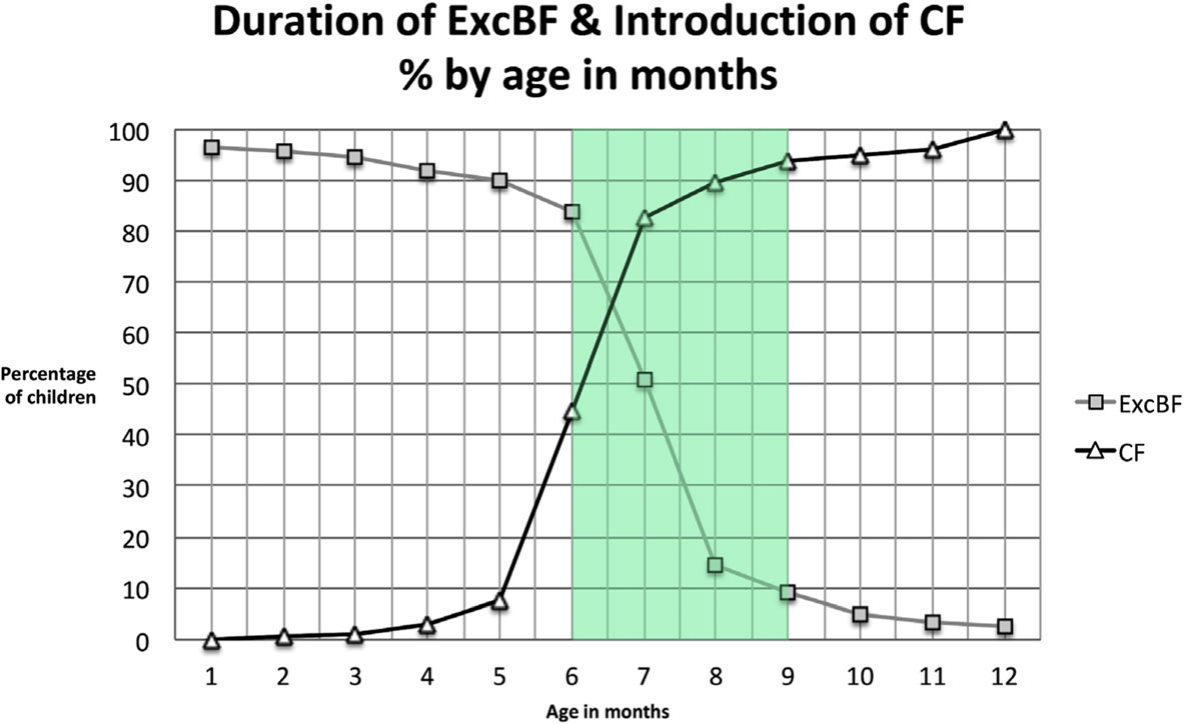
Duration of ExcBF and introduction of CF in percent by age (months). *Note:* CF (*n* = 252), ExcBF (*n* = 231). Timely introduction of CF is highlighted

From the age of 6 months, an infant’s energy and nutrient needs may exceed the energy and nutrients supplied by breastmilk. According to WHO/UNICEF growth faltering may occur when CF are not introduced after a child has completed 6 months of age [23]. The majority (82.1%) of mothers (*n* = 252) introduced solid, semi-solid or soft foods at 6 to 8 months of age, thus matching the proposed indicator for assessing timely introduction of CF [21]. Still 7.6% of mothers introduced CF too early between the 2nd and 5th month, or too late (10.4%) between the 9th to 12th month of age. When relating to the proposed CF indicator then 89.7% of study children have received CF until the age of 6 to 8 months (Figs. 5, and 6).

The reasons for introducing CF at the indicated month of life are specified in Table 4.

**Table 4:** Reason for introducing CF at the respective age

Reason for having introduced CF at the indicated age	Time of introduction of CF	% of total reponses (*n* = 243)
*Family tradition/ Family rule*	*n* = 3 at 5 m, *n* = 50 at 6 m, *n* = 45 at 7 m, *n* = 10 at 8 m to 14 m	44.4
*Child is able to eat*	*n* = 6 at 4 m to 5 m, *n* = 8 at 6 m, *n* = 10 at 7 m, *n* = 1 at 11 m	10.3
*AWC told*	*n* = 1 at 5 m, *n* = 9 at 6 m, *n* = 8 at 7 m to 9 m	7.4
*To promote child growth*	*n* = 2 at 6 m, *n* = 9 at 7 m, *n* = 5 at 8 m to 9 m	6.6
*Everyone says/everyone told*	*n* = 1 at 5 m, *n* = 4 at 6 m, *n* = 4 at 7 m, *n* = 8 at 8 m to 12 m	7.0
*Child needs solid/extra food at this time/ breastmilk insufficient/Child remained hungry after breastfeeding*	*n* = 7 at 6 m, *n* = 2 at 7 m, *n* = 4 at 8 m to 12 m	5.3
*Insufficient breastmilk/Breastmilk decreased/Milk substitute too expensive*	*n* = 5 at 4 m to 5 m, *n* = 4 at 6 m, *n* = 3 at 7 m, *n* = 2 at 9 m to 11 m	5.8
*Subcenter/Doctor told*	*n* = 1 at 6 m, *n* = 5 at 7 m, *n* = 2 at 8 m to 10 m	3.3
*Elder told*	*n* = 1 at 3 m, *n* = 2 at 6 m, *n* = 2 at 7 m, *n* = 1 at 8 m	2.5
*Mother feels it is the appropriate time*	*n* = 1 at 2 m, *n* = 3 at 7 m, *n* = 1 at 8 m	2.1
*Child did not like to eat solid food before*	*n* = 3 at 8 m to 12 m	1.2
*Child starts eating/likes to eat family food*	*n* = 2 at 7 m, *n* = 1 at 10 m	1.2

Minor responses (*n* = 1): *child is able to digest other foods* (at 7 m), *to practice eating solid foods* (at 6 m), *father decided* (at 7 m), *health worker told* (at 7 m), *child needs blood at this age* (at 6 m), *child refused breastmilk at this age* (6 m), *mother moved away and married other man* (at 6 m)

The type of foods for first regular feedings (*n* = 267), were family foods (70.0%), biscuits (30.3%), only rice (15.4%), cereal porridge (3.0%), banana (3.0%), AWC meal (0.7%), roti/chapati (0.4%). When queried for the number of servings of CF on the day before the interview (*n* = 250) the average accounted for 3.5 ± 1.0 times, ranging from 1 to 7 times.

In between the main mealtimes (*n* = 247), almost every fifth (18.2%) mother reported not to offer food to her child, half of all women (49.8%) told to provide foods in between on most of the days, and one third of women (32.0%) said that they sometimes offered food in between. Foods provided in between (*n* = 213), were biscuits (83.6%), family foods (50.7%), plain rice (15.0%), banana (5.2%), puffed rice (2.8%), cake (1.9%), bread (1.4%), cereal porridge (1.4%), lambu (0.5%), chapati (0.5%).

In almost all families (*n* = 266), the child was receiving the same foods at home as the rest of the family (except breastmilk) (97.0%), and there was also no difference (*n* = 287) between the food for the head of the family and that of other family members (99.3%).

### Usual daily feeding routine

The usual number of dry foods/snacks (*n* = 276) was on average 1.4 ± 0.7, ranging from 0 to 4, and the number of cooked meals served over the day (*n* = 276) was on average 2.9 ± 1.1, ranging from 0 to 5. The total number of meals (cooked and/or dry foods) usually offered per day accounted for 4.3 ± 1.4, ranging from 0 to 6.

The majority of families served five meals (63.8%) over the day, followed by four (14.1%) or three (11.6%), and minorities serve 0 to two (8.7%) or six meals (1.8%). Considering age, then the average number of dry/cooked meals served accounted for 0.7 ± 0.9/1.4 ± 1.3 (6 to 8 months), 1.3 ± 1.0/2.0 ± 1.4 (9 to 11 months), 1.4 ± 0.7/3.0 ± 0.9 (12 to 23 months), 1.5 ± 0.6/3.3 ± 0.6 (24 to 35 months), 1.8 ± 0.5/3.3 ± 0.5 (36 to 39 months).

To conclude mothers usually serve three cooked meals and two dry foods (36.6%), or four cooked meals and one dry food (25.0%). Further the serving of three cooked meals and one dry food (9.1%) or two cooked meals and one dry food was common (9.8%). 13.8% of mothers served a different daily meal composition, and 5.8% of mothers did state not to provide any cooked or dry foods to their child even though aged 6 months or older.

Of a maximum of six reported meal times over the day, commonly the first meal of the day was composed of dry foods (57.9%), or only breastmilk/infant formula (21.1%), or breastmilk and dry foods (10.0%), and merely 7.2% reported to serve cooked foods. For all other remaining meal times the serving of cooked foods was more commonly practiced.

### Minimum meal frequency

When relating to the proposed nutrition indicator by WHO/UNICEF [21] for assessing the minimum meal frequency, then more than half of children aged 6 to 8 months (*n* = 28), receive the recommended minimum number of two cooked meals along breastmilk (*n* = 15). Of children aged 9 to 23 months (*n* = 121), 69.4% receive the recommended minimum number of three cooked meals along breastmilk, or 4 cooked meals for non-breastfed children (*n* = 2). To conclude, of all children aged 6 to 23 months, 66.4% receive the minimum number of two to three (four in case of non-breastfed children) servings of cooked food a day.

### Consumption of vegetables, fruits, and animal products during the last 24 h before the interview including foods provided by the government nutrition programs

Fruits were consumed by almost one third (28.8%) of all children (*n* = 274). The type of fruits consumed (*n* = 79), was banana (*n* = 34), jujube (kul) (*n* = 18), guava (*n* = 10), orange (*n* = 5), green mango (*n* = 3), apple (*n* = 3), palm fruit (*n* = 3), grapes (*n* = 2), dates (*n* = 1), lemon (*n* = 1), mango (*n* = 1). Thereby, the majority of children (*n* = 77) ate one kind of fruits, and merely *n* = 2 received two different fruits over the day. The consumption of fruits is not common, and if consumed then according to seasonal availability. The food category “vitamin A rich fruits” (*n* = 274) (including palm fruit or ripe mango) was indicated by 1.5%, and “other fruits” (*n* = 274) by 27.4%.

In the tribal community potato is widely categorized as vegetable. The type of vegetables consumed (*n* = 226), was potato (64.6%), cabbage (18.1%), tomato (15.9%), eggplant (10.2%), cauliflower (9.3%), pumpkin (6.2%), spinach/kulmi (6.1%), mustard leaves (1.8%), radish (0.8%), beans (1.3%), green papaya (0.9%), sorse leaves (0.4%). Of all women (*n* = 275), every tenth (13.8%) provided no potato & no other vegetable to their child during the last 24 h, one fourth (25.1%) provided merely potatoes, and 55.6% offered any vegetables other than potato to their child. Of those receiving vegetables other than potato (*n* = 153), 73.9% received one type of vegetable, 22.9% received two kinds of vegetables and 3.3% received three kinds of vegetables other than potato. The food group (FG) “vitamin A rich vegetables” (*n* = 265) (including green leafy vegetables (GLV), or pumpkin) was consumed by 24.5%, and the group of “other vegetables” (excluding potato) (*n* = 265) by 38.9%.

To conclude during the previous 24 h, “vitamin A rich vegetables and/or fruits” (*n* = 264) were consumed by 26.1%, and “other vegetables (excluding potato) and/or fruits” (*n* = 264) by 58.0%.

With regard to animal products (*n* = 269), the majority of children (78.8%) have consumed any animal products during the previous 24 h. Of those children having received animal products (*n* = 212), about half (56.6%) of children merely received egg, about one fourth (26.9%) received egg and one other animal product, 1.4% received egg and two other animal products. 15.1% received animal products other than egg. Regarding indicated FG consumption (*n* = 269), chicken egg –commonly provided by the AWC, was the most frequently consumed item (66.9%), followed by milk (26.0%), flesh foods like chicken meat or fish (9.7%).

### Dietary diversity -based on child’s vegetable, fruit, and animal product consumption during the last 24 h and current feeding routine of starches and legumes

The daily routine of almost all children (*n* = 276), includes sweets (79.3%) like biscuits, lambu, cake or gur/sugar to sweeten rice; and starchy foods like rice or potato (93.8%). The AWC/ICDS meal was reported to be a fixed part of the child’s day in 87.3% of cases.

Of a total of 264 children (including *n* = 1 non-breastfed), 66.3% have received four or more food groups on the day before the interview (4 FG (40.5%), 5 FG (22.7%), 6 FG (3.0%), median 4 FG, mean ± SD 3.7 ± 1.3 FG, Min/Max: 0/6 FG). When considering the requirements for non-breastfed children, then 65.9% of all children match the minimum dietary diversity indicator. When including merely children aged 6 to 23 months (*n* = 141), then 61.7% matched the recommended dietary diversity of four or more food groups, and 46.8% fulfill both the meal frequency and dietary diversity score (minimum acceptable diet).

### Fulfillment of CF indicators according to sex and age

**Fig. 7:**
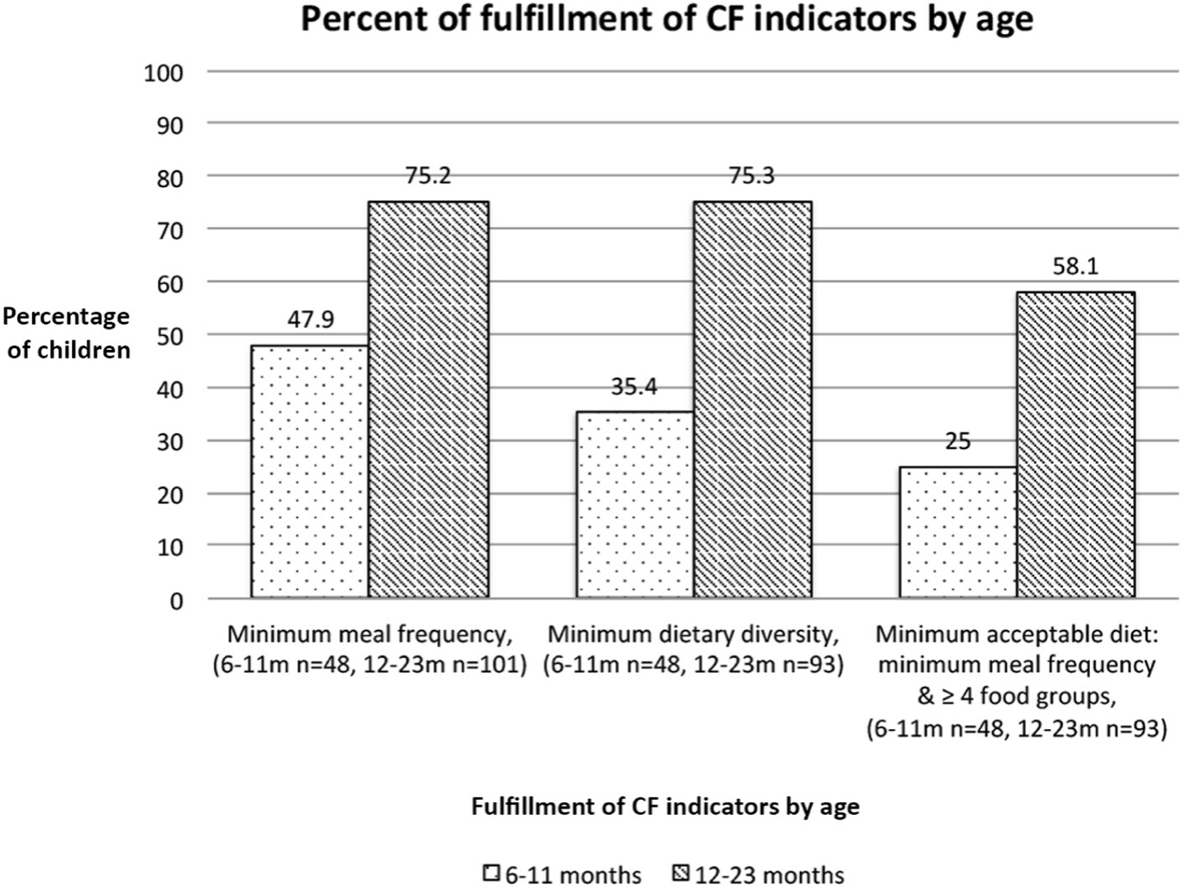
Fulfillment of WHO/UNICEF CF indicators by age for assessing infant and young child feeding practices. *Note: n* = 2 women having a child aged 6-23 m at the time of the interview (presented in Fig. 7 and also in Table 5) did not breastfeed at the time of the interview. Herefore the criteria for non-breastfed children were applied [21]

The fulfillment of dietary CF indicators was similar between both sexes. When judging the CF indicators by age, then indicators on meal frequency and dietary diversity were better matched by the older age group (12 to 23 months) with 58.1% meeting the minimum acceptable diet, versa merely 25.0% of the younger subgroup (6 to 11 months). It has to be noted that in the consideration of nutrition indicators merely study children aged 6 to 23 months were included, as the proposed indicators are targeted at this age range (Table 5, Fig. 7).

**Table 5:** Age-related fulfillment of minimum meal frequency, minimum dietary diversity and minimum acceptable diet by the study children (6 to 23 months)

	*Fulfillment of minimum meal frequency* (definition: 2 cooked meals per day at 6 to 8 months, 3 cooked meals per day at 9 to 23 months)	
	n (%)	N
6 to 8 months	15 (53.6)	28
9 to 11 months	8 (40.0)	20
12 to 23 months	76 (75.2)	101
	*Fulfillment of minimum dietary diversity* (definition: ≥4 FG per day)	
6 to 8 months	7 (25.0)	28
9 to 11 months	10 (50.0)	20
12 to 23 months	70 (75.3)	93
	*Fulfillment of minimum acceptable diet* (definition: minimum meal frequency & minimum dietary diversity)	
6 to 8 months	6 (21.4)	28
9 to 11 months	6 (30.0)	20
12 to 23 months	54 (58.1)	93

Note: for meal frequency: breastfed children (*n* = 147), non-breastfed children (*n* = 2, serial No: 208, 354); for dietary diversity or acceptable diet: non-breastfed child (*n* = 1, serial No 208)

*Recommendation for non-breastfed children:* 4 cooked meals 6 to 23 months (minimum meal frequency), at least 2 milk feeds and *≥ 4 FG* per day not counting milk feeds (minimum dietary diversity)

The count of food groups^NN***^ showed a significant difference (*p<0.001)*   between children aged 6 to 11 months (*n* = 48, median 2 FG, mean ± SD 2.3 ± 1.8 FG, Min/Max: 0/5 FG), and children aged 12 to 23 months (*n* = 93, median 4 FG, mean ± SD 4.0 ± 1.0 FG, Min/Max: 0/6 FG). For upper age categories the mean count of FG was similar or slightly increased (24 to 35 months, *n* = 111, median 4 FG, mean ± SD 4.0 ± 0.9 FG, range: 2–6 FG; or 36 to 39 months, *n* = 12, median 4 FG, mean ± SD 4.2 ± 0.7 FG, range: 3–5 FG).

When considering children having any anthropometric failure (HAZ, WAZ, or WHZ < -2SD) versa non-anthropometric failure, no significant difference regarding the count of FG consumption was detected.

The comparison of children according to their Hb-level showed a significant decrease in the mean count of FG consumed by severity of the level of anemia (*p* = 0.035), hereby the count of FG was significantly different for the groups severe vs. mild anemia (*p* = 0.028), and moderate vs. mild anemia (*p* = 0.010). Non-anemic children consumed a mean of 4.0 ± 1.0 FG (median 4 FG) (*n* = 15), mildly anemic children a mean of 4.2 ± 0.9 FG (median 4 FG) (*n* = 51), moderately anemic children a mean of 3.6 ± 1.4 FG (median 4 FG) (*n* = 185), and severely anemic children a mean of 3.0 ± 1.8 FG (median 4 FG) (*n* = 13). Spearman correlation detected an association between increasing Hb levels and the count of FG consumed (*n* = 264), (*r* = 0.167**, *p* = 0.007). However partial correlation adjusted for age found no significant association (*p* = 0.130) (Table 3).

## Discussion

This study assessed the burden of undernutrition and anemia among Santal mothers and their children and examined feeding and caring practices, which are discussed as drivers of a poor nutritional status in the offspring [24–26].

The burden of child undernutrition –depicted by the mean anthropometric z-score levels and Hb levels, is described in detail in another publication [27].

### Maternal nutritional status and the mother-child dyad

A body of evidence revealed that the maternal nutritional status before and during pregnancy is a critical determinant of birth outcomes, nutritional status and future development of the child [28–32]. In India, intergenerational data of the NFHS-3 (2005-06) [33] revealed that the health, anemia and nutritional status of mother and child are intimately linked and affected by HH wealth or maternal educational level, thus safeguarding a child’s well-being begins with ensuring the health and nutritional status of the mother. Consistent with these findings the present study results suggest an increased risk for the child to suffer from anemia or any anthropometric failure when the mother was affected by anemia or underweight herself. Thus, in order to substantially address child undernutrition, nutrition education of young adolescents including pre-pregnancy counseling, and subsequent promotion of healthy nutrition in the mother-child dyad is pivotal.

The prevalence of maternal anemia (Hb < 12.0 g/dl) and malnutrition (BMI < 18.5 kg/m^2^) among non-pregnant Santal women in the present study highly exceeded regional numbers reported for rural West Bengal in the NFHS-4 (86.2% vs. 64.8, and 49.4% vs. 24.6%) [34], and was still higher than rates reported for females of scheduled tribes (any anemia 75.5%, total underweight 33.2%) [11]. Similarly, according to data examined for the NFHS-3 (national anemia prevalence 55.2% [35], West Bengal 63.2% [34]), the burden of anemia in non-pregnant and non-lactating (NP-NL) women on national level was highest for scheduled castes/tribes (59.1%), with other backward classes (52.5%) or other castes (49.6%) being significantly less affected. Moreover the prevalence and severity of anemia was highest in lactating women, followed by pregnant and then non-pregnant and non-lactating (NP-NL) [36]. As the majority of mothers participating in this study were still breastfeeding the index child at the time of the interview, this may also contribute to the upsetting high rate of anemia.

### Number of children, use of contraceptive methods, antenatal care, child loss and abortion

In this study the use of contraceptives was reported by merely 44.5%, but the time between experienced pregnancies accounted on average for more than 3 years. The NFHS-4 reported the contraceptive prevalence rate with 71%, and 40% of births in West Bengal to occur after 24 months of the previous birth [11].

Lactational amenorrhoea may be relied up to provide 98% contraceptive protection to breastfeeding women during the first 6 months of ExcBF after delivery. Still, continued breastfeeding may have a contraceptive effect from prolonged lactational amenorrhoea up to 1 to 2 years [37]. In 1980, the mean duration of postpartum amenorrhoea in Indian women practicing ExcBF for 8.9 months with continued breastfeeding up to 20.7 months accounted for 11.2 months resulting in an interpregnancy interval of 24.2 months [38]. Similarly, data obtained in the scope of the NFHS-2 (1998-99) for West Bengal indicate a mean postpartum amenorrhea of 10.3 months [39]. The maternal nutritional status was inversely correlated to the duration of lactational amenorrhoea [38, 40, 41], allowing the assumption that in our target group the duration of lactational amenorrhoea is matching or even exceeding that one reported by Prema et al. Still, continued breastfeeding beyond 2 years of child’s age as found in our study, should not be endorsed, as the increasing duration of lactation is associated with a progressive decline in weight-related anthropometric indices in women [38], rather appropriate contraceptive methods should be initiated in time. Moreover continued breastfeeding at 12 to 15 months was associated with a higher risk of underweight in toddlers according to a WHO analysis in 14 low-income countries [42], which may either be the result of reverse causality -with children deemed as more vulnerable being breastfed longer; or the consequence of inappropriate CF practices implied by a one-sided focus on breastfeeding.

The sex ratio among children in this study was 0.872, hereby the number of females per 1000 males is lower than reported for West Bengal in the NFHS-4 with a sex ratio of 0.960 at birth for children born in the last 5 years, or 1.011 for the total population [34]. The child spacing in relation to the use of contraceptives, and sex ratio found in this study may be influenced by multiple aspects: including immutable factors like experienced child loss due to pregnancy complications or an increased risk of morbidity and mortality in the young infant, as well as parenteral exertion of influence in terms of induced abortion directed by the still persisting strong preference for sons in West Bengal but also the beneficial caring capacity of older siblings. Still, the tribal population in India mainly depends upon herbalism to induce abortion for the sake of family planning [43]. The NFHS-4 in West Bengal revealed that 11% of pregnancies terminated in fetal wastage (abortion, miscarriage, or stillbirth) with abortions and miscarriages accounting for 5% respectively. More than one third of abortions (36%) were performed at home. Similarly 11.3% of women in this study indicated to have lost a child before. Abortion was merely pronounced by one woman. As the questionnaire did not explicitly refer to the term abortion, and due to the sensibility of the topic, the occurrence of illegal abortions might be higher. According to quantitative information obtained from a private chamber of a gynecologist in the study area, out of 294 patients investigated from July to December 2014, 35% of patients suffered from medical consequences of incomplete abortions highlighting the relevance of the topic.

Almost all women did make use of ANC visits, however merely 66.3% had their first checkup during the first trimester of pregnancy, and half (53.2%) experienced four or more checkups. In comparison the percentage indicated for scheduled tribes in West Bengal accounted for 53.3 and 79.9% in the NFHS-4, respectively. 89.4% of women had their first ANC visit at the end of the first trimester (3rd month of pregnancy) or at a later stage of pregnancy, which is too late to prevent neural tube defects. The neural tube closure is already completed 28 days after conception, thus folate supplementation is recommended to start already in childbearing age and at least 1 month before conception [44, 45]. The late occurrence of ANC visits may explain partly the high rate of congenital deficiencies in newborns. In India, neural tube defects are reported in 4.5 per 1000 births [46], as compared to an estimation of 1.9 cases per 1000 live births worldwide [47].

In this study the place of delivery was in the majority of cases (69.9%) a health facility, but still one third (29.1%) delivered at home, as opposed to 75.2 and 24.2% as indicated for women in West Bengal, respectively [11]. The assistance during delivery among women in this study versa the NFHS-4 in West Bengal; was done in 71.8% vs. 71.1% by a doctor, in 22.0% vs. 11.3% by a traditional birth attendant, in 14.4% vs. 10.0% by a trained midwife, and in 6.1% vs. 6.5% by friends or relatives. According to the NFHS-4, 28.3% of women did not receive a postnatal checkup. The prevalence of cesarean section was found to be much lower among Santal women (5.7%) as compared to women of West Bengal (23.8%). All in all, the promotion of timely and regular ANC visits as well as assisted deliveries in professional health care facilities including postnatal checkups may avert an increased risk of child loss during pregnancy, delivery, and the first days/weeks after delivery.

On average the mothers had delivered two children at an age of 24.5 years, at the time of the interview. Close to 90% of women and men in West Bengal consider the ideal family size to be two or fewer children. 72% of women who do not wish any more children are already sterilized or have a spouse who is sterilized. The mean number of children born to tribal women in the age of 40 to 49 years accounted for 2.9 in the NFHS-4.

### Breastfeeding practices

The link between malnutrition and infant feeding practices is well established [10, 48].

In this study in the Adivasi villages around Bolpur we found that the majority of mothers (75.7%) initiated breastfeeding in the first hour after birth. Thus, a total of 93.6% have started breastfeeding their newborns in the first 6 h after delivery, and 97.0% within 1 day. These proportions of timely initiation of breastfeeding are higher when compared to the NFHS-4 on scheduled tribes in West Bengal: within 1 h after birth (51.8%), or within 1 day after birth (91.5%) [11]. The percentage and duration of children ever breastfed in our study (99.6%, mean: 35.6 months) is equally higher but similar as compared to the NFHS-4 on scheduled tribes (95.1%, median: ≥36.0 months). According to a study on tribal women in Jharkand merely 29% initiated breastfeeding within 1 h [49], whereas Srikanth et al. reviewed tribals to initiate breastfeeding within the first hour in 16.7% up to 87.1% of cases [50], and a study on tribals in West Bengal reported a proportion of 68.5% to initiate breastfeeding within 1 h after birth [51]. Merely 37.8% of Santals of Orissa were reported to initiate breastfeeding within the first 6 h after birth, but an equally high proportion of 88.6% of breastfeeding mothers reported to feed their baby on demand rather than on schedule (3.8%); as compared to our study with 84.1 and 32.4%, respectively [52].

Pre-lacteal feedings were provided for 6.8% of the newborns, this finding is in line with the NFHS-4 in West Bengal on scheduled tribes (5.1%), and constitute a minor problem among the Adivasis when compared to the total regional level reported at 11.0%. A review on tribals in India found a prevalence of prelacteal feeds ranging from 1.9% up to 68.0% [50].

In West Bengal 52.3% of children aged younger than 6 months are exclusively breastfed, vice versa almost half of mothers with children < 6 months is not practicing ExcBF when queried for the current breastfeeding status [11]. Similarly a study on Santal women in Burdwan District of West Bengal reported a current ExcBF rate in children under 6 months of 46.2% [53], and a study on tribal women in Jharkand found an ExcBF rate under 6 months of 47% among the proportion of study children aged 0 to 5 months [49]. In our study (*n* = 267), the majority of mothers (76.8%) indicated to have performed ExcBF for any period of time. Thereof 16.1% of mothers retrospectively reported having performed ExcBF for less than 6 months, 74.9% for 6 to 8 months, and 9.1% for 9 up to 12 months. In the NFHS-4 of West Bengal [11] exclusive breastfeeding during the previous 24 h (current ExcBF status) applied to 13.9% of children aged 6 to 8 months, 6.5% of children aged 9 to 11 months, 5.3 and 4.0% for those aged 12 to 15 months or even 12 to 23 months, respectively. Altogether the ExcBF rates were much higher in our study, which may be attributable to recall bias as data were obtained retrospectively by recalling ExcBF durations foremost relating to the distant past. The indicated median duration of ExcBF accounted for 7 months as opposed to merely 3.5 months in the NFHS-4 for scheduled tribes. The median duration of predominant breastfeeding was indicated with 6.8 months in the NFHS-4 of West Bengal [11]. In our study the provision of plain water or candy water during the first 6 months of child’s life in addition to breastmilk was mentioned by 6.0% of mothers, formula milk or cow milk by 13.5%, and CF by 8.2%, as opposed to 18, 11% or 10%, respectively in the NFHS-4, India. It has to be noted that equally the current breastfeeding status during the previous 48 h, as well as long-term recall data on ExcBF are reported not to accurately reflect the feeding pattern since birth [54].

Perceived insufficiency of breastmilk was mentioned by 13 women (4.6%) as reason for making use of other milk substitutes in addition to breastmilk, similarly 5.7% named insufficient breastmilk as decisive point of introducing CF. Consistent with this finding perceived insufficiency of milk was a major reason for non-exclusive breastfeeding, or delayed initiation of breastfeeding in other Indian studies [55–57], and has been also shown to be a major reason for low ExcBF rates among Chinese mothers [58].

In our study one third of mothers (32%) discarded the colostrum. Major reasons were the adherence to traditional beliefs either taking shape through the exerted influence of elders or the instilled belief of the first milk being too sticky or thick. Most striking however was the fact that women even were prompted by doctors and nurses to deprive their child of the colostrum, highlighting the necessity to promote proper breastfeeding policies in the health care sector as well as the general public. A review including 17 Indian studies found a huge variation in the feeding practice of colostrum to newborns, with colostrum being discarded by 1.6% up to 65.0% of mothers. Similarly the elder’s advice and perceived harmfulness of colostrum were crucial in the decision-making of discarding the “golden milk” [56]. Equally another review including 11 quantitative studies on tribals in India reported discard rates of 1.0% up to 60.7%, with the author correctly pointing out the perceived lack of awareness about the benefits of colostrum (that it is rich in nutrients and protective antibodies), as opposed to the willing denial of feeding due to tradition such as elder’s advice or misconceptions like “it is impure, cheesy, not good for health, not easy digestible, or causes diarrhea” [50]. A study on tribal women (Santal, Oraon, Mahli, Ho) of Jharkhand found colostrum to be discarded in one third of cases (36%) [49]. Colostrum feeding practices in Assam on rural women revealed a discard rate of 29.5%. Of those discarding the colostrum the major cited reason was adherence of elder’s/relatives advice, followed by the believe the child could not digest colostrum, that it is bad milk, or ignorance of the mother [59]. A study from Central India reported discard rates of 23% and came to the conclusion, that although breastfeeding is practiced universally, there is the need for closing the gap between knowledge and practices (e.g. ExcBF rates, colostrum feeding, and the duration of breastfeeding sessions have to be increased, and swapping the breasts during one episode of feeding or decreased feeding during child’s illness have to be averted) [60].

### Complementary feeding practices

When relating to the proposed CF indicators by WHO/UNICEF for children aged 6 to 23 months, then in our study merely 10.3% of children did not receive CF when aged 6 to 8 months, 33.6% did not match the recommended minimum number of servings of CF, and an inadequate dietary diversity (< 4 food groups/day) was found in 38.3%, as the AWC meal is commonly included in the daily routine. Altogether out of 141 children, 46.8% obtained an acceptable diet meeting both, the minimum meal frequency and minimum dietary diversity. This prevalence is more satisfying as compared to the NFHS-4 in West Bengal, reporting 48% of children not being offered food until age of 6 to 8 months, 62% of children (breastfed and non-breastfed) not receiving the minimum number of feedings or the appropriate dietary diversity, respectively. In regard to scheduled tribes in West Bengal (breastfed children) then the minimum meal frequency was not achieved in 57.9% of children, and 64.8% did receive less than four food groups, thereby both indicators were met by merely 20.2% [11]. Equally more than half of Santal mothers (53.3%) did not start CF in time, did not provide the minimum meal frequency (58.5%), or the minimum dietary diversity (69.2%) [53]. More satisfying rates were achieved by a study on feeding practices in rural North India. Here merely 10.7% of children aged 6 to 8 months have not yet been introduced solid or semi-solid foods, and 6.9% of breastfed infants (6 to 23 months) did not match the minimum meal frequency. Of children aged 6 to 23 months, 13% did not receive the minimum dietary diversity, and according to age 22.4% (6 to 11 months), 9.9% (12 to 17 months), and 7.2% (18 to 23 months), respectively were deprived of the minimum dietary diversity. Altogether of all children aged 6 to 23 months, the majority 86.2% was offered the minimum acceptable diet [57]. Equally, findings of our study and an analysis on 14 low-income countries found the minimum acceptable diet to increase by age [42].

However in regard to our study it remains to be emphasized that the provision of AWC/ICDS meals is the only potential source of cow milk/egg as merely named by 3.3%/ 1.8% of mothers (*n* = 276) respectively to be common foods prepared for the child at home. Similarly, the provision of dal to infants and young children remains improvable, as the home-based preparation of legumes for the child’s diet was performed at maximum by 29.0% of mothers that stated “family foods” or “dal” to be common foods offered to the child. When not considering the AWC/ICDS meal, the serving of plain rice over the day without family foods, potatos, dal, fish or vegetables was practiced by every third mother (34.4%), remaining the only home-prepared food offered aside biscuits/cakes (which too often serve as first CF) and/or milk/breastmilk. In accordance food frequency data on *n* = 31 Santal children (6 to 36 months) obtained in the scope of a pre-study in 2014, revealed the majority of mothers not having prepared fruits (*n* = 23), dairy products (*n* = 25), egg (*n* = 23), meat (*n* = 17), respectively, for their child on household level during the preceding 7 days. Fish or snails, were consumed once a week up to every second day by the majority of cases (*n* = 21). At least every second day the serving of pulses/legumes (*n* = 15) and vegetables (*n* = 24) was practiced, whilst cereals, starchy roots, sugars, and oil were integrated in the daily diet for all children except one. Quantity analysis including the AWC/ICDS meal revealed an average daily consumption of 87 g cereals (raw amount), 30 g biscuits/sweets/sugar, 23 g starchy roots, 22 g colored vegetables, 14 g dairy products, 10 g pulses and legumes (raw amount), 8 g egg/chicken/meat/fish, 4 g fruits, 3 g oil [61], invariably falling below recommendations for all food groups except cereals and sugar [62], highlighting a grossly inadequate quantity of CF in regard to different types of food groups offered to children.

When assessing the fulfillment of CF indicators by age, children younger than 1 year have had a poorer outcome in meal frequency and dietary diversity, highlighting the risk exposure of that vulnerable subgroup. This risk especially becomes apparent when considering that family foods (spicy, hard consistency) or plain rice, biscuits (low nutrient density) were major types of foods provided at household level. Similarly, the feeding of household diets to young children was found to be common in many low- and middle income countries. Maternal and child dietary diversity were associated with agreement for staple foods, but disagreement in vegetables, dairy products or flesh foods, as children were fed merely a subset of family foods [63]. Moreover preceding food weighing records on Santal children 6 to 11 months (*n* = 6) and 12 to 24 months (*n* = 14), revealed the daily nutrient intake by CF not reaching the 30 and 50% mark of Indian RDA [64] for all micronutrients and the vast majority of macronutrients, respectively. Even when assuming a medium breastfeeding frequency for both age groups, in particular the provision of calcium, iron and zinc continued to fell below 50% of RDA [61]. Inappropriate feedings undermine adequate intake in the child quality and quantity wise. Mothers have to be counseled about the necessity to introduce adequate food starting from 6 months, and to appropriately increase diversity and quantity soon after. Data of our baseline assessment revealed anemia to be more prevalent among children under 24 months than among older study children [27]. This observation is in accordance with the NFHS-4 West Bengal [11]. Amongst other reasons the improved ability of the child to eat family foods by increasing age may be decisive for the gradual rise of the Hb levels achieved during the second year of child’s life. Interactive cooking trainings on household level by trained community workers may give mothers the understanding of appropriate infant foods like porridges, that are easily to swallow by the infant. The teaching of how to prepare an instant mix porridge enriched with oil, sugar, milk powder, vegetables and fruits may unburden mothers in regard of time constraints [65].

Moreover even though the AWC/ICDS meal has been reported to be an integral part of daily feeding routine and constitutes a decisive dietary enrichment, on-sight observations correspond with reported mismatches between the ICDS program’s design and actual implementation [66, 67] e.g. leakage in the take-home food ration as intra-household sharing is commonly practiced [61], thus the full potential of the supplementary meal may not be achieved for the target child. Consequently the presumed more satisfying dietary diversity resulting from the AWC/ICDS meal may be misleading, due to diminished quantities of key foods being utilized by the index child alone. Further irregular program delivery or unstable distribution of egg is reported, as possible reason why the ICDS remained unsuccessful in reducing stunting rates [68]. Similarly obtained qualitative information in two Centers in the project area already indicated that the provided full egg is sometimes replaced by half of that portion, and amounts of oil, vegetables, potatoes and soy allocated per child varied across the Centers showing several limitations in the performance of the ICDS authorities. Further, the money provided per beneficiary is not directly linked with consumer price index rather is allocated by a fixed budget per interval, thus cannot offset inflation implying variations in the quantity of food provision [69]. Moreover it has to be considered that even though providing severely malnourished children with higher food quantity the nutrient and energy density of the AWC meals is not matching the reference nutrient intake values for malnourished children. The nutritional status may not be used as indicator of ICDS program performance [70], but still recent program evaluations reported the ICDS program –launched in 1975, having had a positive impact on normal children and those suffering from a mild form of malnutrition, whereas there was no impact on children suffering from moderate or severe forms of malnutrition [71]. According to Dutta et al. increased supervision but also intensified complementary nutritional education will maximize the impact of ICDS services on child growth [68]. AWC worker may be further encouraged and made aware of the importance of their work to raise more awareness about the necessity of proper nutrition for the whole family. Despite the named deficits, the ICDS programs constitute a huge benefit for the tribal communities by making animal products accessible and providing a warm meal to the child.

In this study an increased HH cash income was associated with shorter duration of ExcBF, as well as a timely introduction of CF. Similarly, based on a secondary analysis of the NFHS-3, Patel et al. reported wealthier HH to be less likely to delay introduction of CF [72], and reported richer HH to have a lower prevalence of ExcBF (however in our study the reported prevalence of ExcBF was balanced between both income groups). Moreover economic, educational and cultural aspects are discussed to be major constraints on the way to increase dietary diversity [63, 73]. In our study no quantitative associations between economic aspects or maternal educational level and the fulfillment of minimum acceptable diet could be detected. Still 92.7% of mothers (*n* = 281) felt that the regular consumption of vegetables or fruits is important, but stated as major reasons for not increasing the consumption (*n* = 262): limited purchasing power (82.1%) or limited availability nearby home (17.2%), [27]; altogether stressing the importance of strengthening income and reducing market distances for an increased accessibility of diversified diets.

Infant feeding goes beyond the nutritional [74] and economic aspects. Responsive feeding is not commonly practiced among Santal mothers, as not being culturally integrated in mother-child care. On-sight observations show that the mother uses to sit behind her child, thereby maintaining little eye-to-eye contact. Thus, even if the mother sits close to the child, there is much improvement and behavioral change needed for proper motivation of the child to eat. Frequently Adivasi women tell during the consultation hour in the outpatient department of the St. Mary’s Child and Mother Health Care Centre, that their child is only liking rice. However, when offering spontaneously a banana or after preparing some nutrient-rich and energy-dense porridge at the Centre, the child happily accepts the food. Thus, mothers have to learn how to prepare proper food suitable for infants, further to be aware that the rejection of some food should not be acquiesced as justification for not offering other types of food to the child. A review on complementary feeding patterns in India suggested that the perception of child’s hunger might play a major role in poor CF practices e.g. mothers being likely not to feed their child until the child showed hunger. However increased severity of undernutrition implies loss of appetite fueling the vicious cycle of malnutrition. The authors concluded detrimental feeding practices to be linked to poor socio-economic status, low maternal educational level, adverse socio-cultural beliefs, and ignorance. Not unless mothers are successfully counseled, progress achieved by interventions will continue to be modest [75].

### Use of time on child care

The invested time on child care accounted for 2.9 h per day. This limited time for child care even exceeds the one indicated by other studies from Nias Island (2.1 h) [76], Mozambique (0.4 h) [77], or Rajasthan (2.5 h care for children, elderly and disabled) [78]. Cooking related activities consume most available time of the mother (7.8 h). The preparation of the lunch requires the only clay fire cooking zone already in the very early morning (merely 1.9% of *n* = 265 families reported to own a gas cooker), thus due to practical reasons the child’s breakfast constitutes foremost of ready-to-serve dry foods and snacks. Food and food habits are an integral part of culture and living circumstances. When aiming for sustainable nutrition awareness, any aspired changes of cooking behavior should be integrated in and inspired by daily routine and food habits. Modifying a few components within the scope of cultural habits will be more fruitful, as opposed to inventing new structures that will easily fall in oblivion soon after, due to inconvenience. Therefore when seeking an improvement in the nutrition quality towards five nutritious meal times, the breakfast may be exemplary supplemented with a variety of ready-to serve fruits and if available a glass of milk, the AWC meal may be quickly enriched with one to two tablespoons oil and lemon juice by the mothers themselves at home, whereas the family lunch better presents itself for a more holistic modification in food quality and quantity according to the widely counseled five food groups –placing priority on increased amounts of vegetables other than potato and legumes, the snack in the afternoon should be composed of an adequate quantity of fruits or nuts (instead of commercial products like biscuits or puffed rice), and the family dinner -which is often served too late for the young children, may be served earlier and be composed of a nutritious instant porridge. The promotion of family kitchen gardens is pivotal when seeking to provide children with an adequate quantity of fruits and vegetables [79]. Presently traditional porridges served to the children, tend to be composed of a cereal component with sugar, rarely including milk powder, thereby lacking legumes, fruits, vegetables and oil.

A huge gender division of labor over the day has been shown in Mozambique, with men spending an average of 6.4 h per day on daily work activities versa 11.7 h spent by women [77]. Women’s responsibilities mainly embrace reproductive activities including child bearing and rearing, household maintenance, and caring for elderly and sick, but also income generating activities or family subsistence farming, and social and community-building activities. However, if women’s work burden is reduced they tend to invest the free time in many different ways such as additional caring and domestic works, income generating activities, education, or political engagement [80], and less likely for community or social activities, and leisure [81]. Also the definition of leisure time is varying, e.g. in India “free time is considered as leisure so long as the woman can sit or remain stationary, even if mending clothes”, whereas in Egypt even social interaction is not perceived suchlike [80]. Anyhow, especially the indicated time use on child care remains low across studies, and according to own observations child care is often restricted to the very essential caring activities and lacking playful stimulation and interaction.

Overall, tribal mothers residing in the villages surrounding Bolpur are burdened with a high daily work load, mainly dominated by efforts to satisfy the family’s needs, thereby women are usually engaged in field works or income-generating activities aside pursuing core cooking activities, and domestic works. According to own observations women are already fully engaged in works other than child care, but this condition is perceived as normal, and child care runs simply along the daily work routine. Thus, even though the majority of mothers reported to have sufficient time to complete all works and to care for the child at the same time, it has to be considered that a typical child care routine, with the child being in the focus of care is not culturally familiar. The harsh living conditions make child caring capacities limited amongst the majority of Santal women and are a contributing factor why mothers as well as their children eventually find themselves undernourished.

Thus, the whole family should become more conscious in jointly placing priority on proper supervision, care, physical, cognitive and socio-emotional stimulation of their children. More than 200 million children below 5 years of age do not achieve their full potential due to poverty. In the circle of the intergenerational transmission of poverty these children are likely to have a poor nutrition and health status, have poor school achievements, subsequently low incomes, high fertility and due to lack of education or experienced stress/depression low responsivity for their children resulting in deficient child care and stimulation at home, respectively [82]. Work participation of Indian women resulted in less time spent on child care (playing activities), leisure time and personal care activity [83]. Similarly, in our study a higher HH cash income was associated with less time invested on caring activities, possibly being associated with the involvement of the mother in income producing activities. Women in Rajasthan were found to spend 9.5 h on unpaid domestic or agricultural work and merely 17 min on paid work [78]. Policies need to consider women’s time constraints and use, to promote technology, services, awareness building, and infrastructure in a gender-sensitive way to lower the female burden of domestic activities, productive work and overall care. Then, women may better focus on their children with greater men’s involvement in all spheres of action, and less involvement of older siblings as childcare provider. Recently the government introduced a free cooking gas scheme and provided gas cookers to poor families, which is one important step to reduce the time constraint of mothers and making the preparation of special infant foods like porridges more feasible. Childcare cooperatives may unify the access to adequate promotion of the child’s development and empower mothers to more efficiently complete daily works, with the option to increasingly pursue paid work and subsequently provide a better quality of care to their children.

### Limitations

Variant types of anemia with different etiologies could be a major confounding factor for the current study. Recall bias may still be present even though social workers were trained to minimize this confounding factor commonly applying to retrospective studies including self-reporting as primary data source. In particular recall bias may apply to events in the distant past e.g. prelacteal feeding, initiation of breastfeeding, or performance of ExcBF. Long-term ExcBF recall tends to overestimate the ExcBF duration, thus feeding data should ideally be collected prospectively [54]. In the current study, reporting bias additionally arises as merely the mother herself was asked –who however is often weak or even separated at some time from her baby after delivery, thus may not be aware of all activities family members have performed. Consequently, the additional interviewing of relatives (e.g. grandmother) would bring more precise information on prelacteal feeds or the initiation of breastfeeding but was not possible to be obtained in the scope of this study. As the analysis of the minimum dietary diversity was rather based on the recalled feeding routine for starches and legumes than the actual feeding practice during the previous 24 h, a slight overrating may be the case in regard to the assessed feeding of these two food groups (still it has to be considered that starch/lentil-containing AWC/ICDS meals are available 6 days a week, thus extensive overestimation is not assumed). Moreover, general limitations in regard to sample size apply to this study, e.g., not being powered for all baseline characteristics presented, as the sample size was powered for the intervention purpose.

## Conclusion and recommendations

In summary, the present study revealed a high prevalence of undernutrition and anemia among Adivasi mothers and their index child. Suboptimal child feeding and caring practices were identified. Still, as compared to national data, indicators for proper infant feeding practices were fulfilled by a higher proportion. However children aged 6 to 11 months were particularly vulnerable with merely every fourth child fulfilling the minimum acceptable diet, and being commonly served household diets instead of specially prepared infant foods. Mother-child dyad analysis stressed the importance of focusing on interventions embracing both mother and child, in order to break through the intergenerational cycle of malnutrition. The supplementary AWC meal definitely increased children’s meal frequency and dietary diversity. However on HH basis the serving of animal-sourced foods, legumes, vegetables and fruits has to be scaled up. Communal home garden activities or crop diversification may be a cost-effective measure to improve IYCF practices, as purchasing power was associated with timely introduction of CF and as due to limited market/cash availability mothers refrain from increasing their vegetable and fruit consumption. Thus, kitchen gardens may be a sustainable local measure compensatory for distanced markets along with the potential of generating income. Educational interventions have to raise awareness about traditional myths related to infant feeding practices, increase maternal nutrition knowledge for specially prepared infant foods (quality and quantity-wise), and aim for the adoption of responsive feeding strategies by the mother. In particular, village health workers have to increase the awareness regarding the benefits of feeding colostrum and appropriate infant feeding choices for the offspring. Then mothers will be able to best use their limited resources to enhance the health and nutritional status of their children. Moreover maternal self-confidence in breastfeeding needs to be strengthened, in order that any perceived insufficiency in breastmilk is not handled by decreasing the breastfeeding frequency through introduction of breastmilk substitutes. Breastfeeding promotion should focus on the general public, thereby equally targeting all community members as well as the health care sector. Moreover women need to be supported to make regular use of contraceptive methods in order to allow mothers to control child spacing, thereby averting the perceived high rate of abortions among the tribal community.

Altogether this study provided a range of anthropometric, hematological and nutrition-related background information on Santal mother-child dyads, constituting possible new entry points for future intervention activities.

## Data Availability

The datasets used and/or analyzed during the current study are available from the corresponding author on reasonable request.
